# Spatiotemporal Transcriptomic Atlas Reveals the Regulatory Mechanisms Underlying Early Inflorescence Development and Sex Differentiation in Spinach

**DOI:** 10.1002/advs.202507818

**Published:** 2025-06-26

**Authors:** Chen You, Hao Yang, Yueyan Zhao, Xiaoning Wang, Shuaijie Wei, Ning Chen, Yulan Zhang, Luxian Liu, Wei Qian, Shufen Li, Wujun Gao

**Affiliations:** ^1^ College of Life Sciences Henan Normal University Xinxiang 453007 China; ^2^ College of Life Sciences Henan Agricultural University Zhengzhou 450002 China; ^3^ State Key Laboratory of High‐Efficiency Production of Wheat‐Maize Double Cropping Center for Crop Genome Engineering College of Agronomy Henan Agricultural University Zhengzhou 450046 China; ^4^ State Key Laboratory of Vegetable Biobreeding Institute of Vegetables and Flowers Chinese Academy of Agricultural Sciences Beijing 100081 China; ^5^ Zhongyuan Research Center Chinese Academy of Agricultural Sciences Xinxiang 453519 China

**Keywords:** dioecious plant, epigenetics, inflorescence development, MSI1, sex differentiation, spatial transcriptomics, spinach

## Abstract

Dioecious plant species produce male and female flowers on separate individuals. However, the molecular regulatory mechanisms underlying these processes remain poorly understood. In this study, the model dioecious spinach (*Spinacia oleracea*), a widely cultivated leafy vegetable, is investigated by analyzing spatial transcriptomes of the shoot apices at different developmental stages. The findings elucidate key regulatory pathways underlying both inflorescence development and sex differentiation. Through the characterization of dynamic transcriptional changes across four consecutive developmental phases, distinct cell types within the shoot apex and stage‐specific marker genes are identified. Notably, pseudotime trajectory analysis reveals that spinach sex differentiation occurs at the four‐leaf stage. Furthermore, the critical involvement of epigenetic mechanisms in this process is demonstrated. By integrating functional genetic validation, the epigenetic regulator MULTICOPY SUPPRESSOR OF IRA 1 (MSI1) together with histone deacetylase HDT2 is discovered to promote female differentiation. Collectively, this work provides comprehensive insights into the regulatory networks controlling inflorescence development and sexual differentiation in spinach and establishes a valuable resource for advancing sex differentiation research in dioecious plants.

## Graphical abstract

High‐resolution spatiotemporal transcriptomic data from male and female spinach inflorescences across four key stages first reveal the sex differentiation initiates at the four‐leaf stage and is governed by epigenetic regulation via the SpMSI1‐SpHDT2 complex. Moreover, this spatiotemporal atlas provides a valuable resource for further dissecting the molecular regulatory mechanisms underlying flower development in dioecious plants.

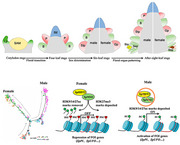

## 1 Introduction

The majority of flowering plants, or angiosperms, possess bisexual flowers that contain both fertile stamens and pistils within a single flower. In contrast, ≈6% of flowering plants exhibit a dioecious reproductive strategy, characterized by the presence of separate male and female flowers on distinct individual plants.^[^ ^1^ ^]^ Similarly, ≈6% of flowering plants are monoecious, featuring both male and female flowers on the same plant but in separate floral structures.^[^ ^2^ ^]^ Both dioecious and monoecious plants produce unisexual flowers. In the context of angiosperm reproduction, the bisexual system is regarded as the ancestral sexual form in angiosperms, while the emergence of unisexual systems represents a significant evolutionary transition.^[^ ^3^ ^]^ Among these unisexual plant species, many hold economic and scientific importance, such as monoecious cucumber (*Cucumis sativus*) and maize (*Zea mays*), as well as dioecious spinach (*Spinacia oleracea*), garden asparagus (*Asparagus officinalis*) and papaya (*Carica papaya*).^[^ ^4^, ^5^, ^6^ ^]^ Therefore, studying the development of unisexual flowers not only elucidates the mechanisms underlying plant sex determination and differentiation but also provides fundamental theoretical knowledge for the hybrid breeding of these economically important plant species.

Currently, the developmental processes of bisexual flowers are relatively well understood. It has been established that bisexual floral organs consist of four whorls, with their development regulated by the ABC model of gene expression. Specifically, the first whorl (sepals) is governed by A‐class genes, the second whorl (petals) by A‐ and B‐class genes, the third whorl (stamens) by B‐ and C‐class genes, and the fourth whorl (carpels) exclusively by C‐class genes. This model has been revised and expanded into the ABC(D)E model in recent studies.^[^ ^7^ ^]^ By contrast, the mechanisms of sex differentiation in unisexual flowers are more complicated. The development of unisexual flowers has arisen independently multiple times in both dioecious and monoecious plant taxa.^[^ ^8^ ^]^ Based on ontogenetic processes that give rise to unisexual flowers, two principal pathways have been proposed: 1) Type I pathway: Flowers initially develop as bisexual but become unisexual through the developmental arrest of either the gynoecium or androecium in later stages; 2) Type II pathway: Flowers are unisexual from the outset, with sex differentiation occurring at a very early stage.^[^ ^9^ ^]^ Even within Type I flowers, the timing of gynoecium or androecium abortion varies significantly among plant species, corresponding to their repeated evolutionary origins.^[^ ^8^ ^]^ Consequently, unisexual flower development is hypothesized to involve diverse genetic and developmental mechanisms. Contemporary research suggests the involvement of complex regulatory networks, including multiple genes, transcription factors (TFs), and epigenetic modifications.^[^ ^10^, ^11^, ^12^, ^13^ ^]^ However, the detailed mechanisms governing sex differentiation, particularly the regulatory patterns during the early developmental stages of primordium differentiation in dioecious plants, remain poorly understood.

Comparative transcriptome profiling of male and female flowers in dioecious plants is an effective approach to identify candidate genes implicated in sex differentiation.^[^ ^14^, ^15^ ^]^ For example, transcriptomic analysis of early‐stage male and female flowers in papaya has revealed differentially expressed genes (DEGs) associated with sex differentiation, with functional annotations highlighting roles in transcriptional regulation, epigenetic modification, and phytohormone signaling.^[^ ^13^ ^]^ However, traditional transcriptome sequencing is limited by tissue heterogeneity and the lack of spatial resolution in gene expression data, which hampers the precise elucidation of the mechanisms underlying sex differentiation in dioecious plants. Recent advances in spatial transcriptomics overcome these limitations by enabling in situ spatial gene expression profiling, allowing for precise mapping of transcriptional activity within intact tissues. These techniques have elucidated molecular mechanisms underlying diverse plant developmental processes, including nitrogen fixation and nodule development in soybean,^[^ ^16^ ^]^ seed germination in barley,^[^ ^17^ ^]^ shoot regeneration in tomato callus,^[^ ^18^ ^]^ stem development in poplar,^[^ ^19^ ^]^ and rapid shoot growth in bamboo.^[^ ^20^ ^]^ Particularly, recent studies in orchids have demonstrated the effectiveness of spatial transcriptomics in unraveling the mechanisms of floral organogenesis. This approach enables the quantitative mapping of spatial gene expression patterns, thereby linking morphological changes during flower development with genome‐wide molecular profiles.^[^ ^21^ ^]^ Such technological advancements offer significant potential for systematically dissecting molecular and cellular mechanisms underlying early sex differentiation in dioecious plant species.

Spinach (2n = 2x = 12) is one of the most widely cultivated high‐nutrition leafy vegetables in the world.^[^ ^22^ ^]^ As a dioecious species, its gender determination is governed by sex chromosomes. The X and Y chromosomes are cytogenetically homomorphic, with the Y chromosome containing a relatively small sex‐linked region of 24.1 Mb, indicating a recent evolutionary origin of dioecy.^[^ ^23^ ^]^ The spinach flower exemplifies a Type II flower, following the “unisexual from inception” developmental pathway. The totipotent meristematic cells undergo a brief vegetative phase before transitioning to the reproductive stage for floral initiation. During this developmental shift, the vegetative shoot apical meristem (SAM) differentiates into an inflorescence meristem (IM), which produces lateral meristems containing both flower primordia (P) and bract primordia (B). These flower primordia ultimately develop into unisexual male or female flowers. Notably, the sexual fate of developing flowers can be modulated by environmental cues, including exogenous phytohormones and elevated temperatures, suggesting plasticity in spinach's sex differentiation mechanisms.^[^ ^24^, ^25^ ^]^ Given its short life cycle under controlled laboratory conditions and the availability of multiple high‐quality genome assemblies for cultivated varieties, spinach has emerged as a premier model system for investigating the molecular mechanisms underlying inflorescence development and sex differentiation.^[^ ^22^, ^23^, ^26^, ^27^ ^]^ Several genes involved in floral development have been characterized, including *SpAP1‐1/2*, *SpFUL*, *SpAP3*, and *SpPI*.^[^ ^28^, ^29^, ^30^ ^]^ In addition, recent transcriptomic analyses have identified numerous DEGs associated with TFs and phytohormone signaling pathways in male versus female flowers.^[^ ^31^, ^32^ ^]^ However, these genes primarily regulate floral organ identity, and the molecular mechanisms underlying sex differentiation, particularly during the early stages of flower development, remain largely unexplored.

In this study, we present a developmental‐series spatial transcriptomic analysis of male and female spinach shoot apices, encompassing floral primordia and early inflorescence. The dynamic expression patterns of genes and transcriptional networks of male and female shoot apices at various developmental stages provide a spatiotemporal atlas of floral meristem initiation and early floral organ development. By integrating genetic experimental validation, we uncover novel insights into early sex differentiation mechanisms in spinach, with an emphasis on epigenetic regulation. These findings elucidate the intricate interplay between cellular differentiation and sex differentiation in spinach, offering valuable resources for further genetic studies on sex differentiation mechanisms in spinach and other dioecious plants.

## 2 Results

### 2.1 Morphological and Structural Analysis of the Vegetative‐To‐Reproductive Transition in Spinach

**Figure 1:**
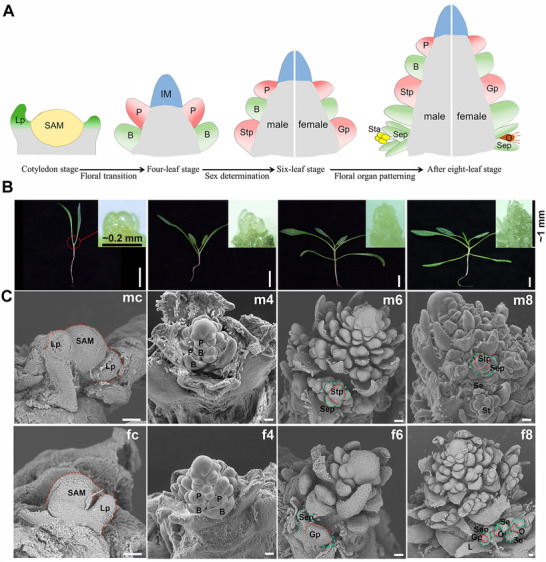
The developmental process of spinach inflorescence and morphological observation by SEM. A) A schematic diagram of early inflorescence development in spinach from meristem to early floral organ specification, including three key biological processes: floral transition, sex differentiation, and floral organ patterning. SAM, shoot apical meristem; Lp, leaf primordium; IM, inflorescence meristem; P, flower primordia; B, bract; Stp, stamen primordia; Gp, gynoecium primordia; Sta, stamen; O, ovule; Sep, sepal. B) Representative images of developing male inflorescences sampled at four key developmental stages. White scale bars, 2 cm. C) Scanning electron microscopy reveals distinct morphological characteristics between developing male and female inflorescence. White scale bars, 100 µm.

To investigate the dynamic morphological changes during the transition from vegetative to reproductive growth in spinach, we performed scanning electron microscopy (SEM) on the developing male and female inflorescences across stages ranging from the cotyledon stage four‐leaf, six‐leaf, eight‐leaf, ten‐leaf, and twelve‐leaf stages, up to the fourteen‐leaf stages (bolting) (Figures S1 and S2, Supporting Information). Among these, the cotyledon (male cotyledon, MC; female cotyledon, FC), four‐leaf (M4/F4), six‐leaf (M6/F6) and eight‐leaf (M8/F8) stages represent key consecutive transitional phases in inflorescence development, during which the developmental trajectory progresses from the SAM to the IM, and then to the P, ultimately leading to the formation of distinct male and female floral organs (**Figure** **1**A,B). In detail, at the cotyledon stage, a single SAM enclosed by two leaf primordia (Lp) is present at the stem apex, remaining in a vegetative state with no visible reproductive structures. During the four‐leaf stage, the SAM transitions into a dome‐shaped inflorescence meristem (IM), accompanied by the emergence of several floral primordia and their subtending bracts (B) around the IM periphery, marking the onset of floral transition. At both stages, no discernible morphological differences are observed between male and female samples. The first noticeable distinction appears at the six‐leaf stage, marked by sepal emergence: four sepals differentiated from the base of the male flower primordia, whereas only two develop in the female floral primordia, indicating the occurrence of sex differentiation. At this stage, stamen primordia (Stp) form in a phyllotaxic pattern around the IM in males, while gynoecium primordia (Gp) initiate in females. By the eight‐leaf stage, distinct male and female floral organs are clearly differentiated, completing the early floral organ patterning phase, with the Stp expanding to form stamens (St) and the Gp developing into ovules (O) (Figure 1C). Collectively, these four stages capture the early inflorescence developmental stages from meristem transition to floral organ emergence, encompassing three key biological processes: floral transition, sex differentiation, and floral organ patterning.

### 2.2 Construction of a Spatial Transcriptome (ST) Atlas of the Early Inflorescence Development in Spinach

**Figure 2:**
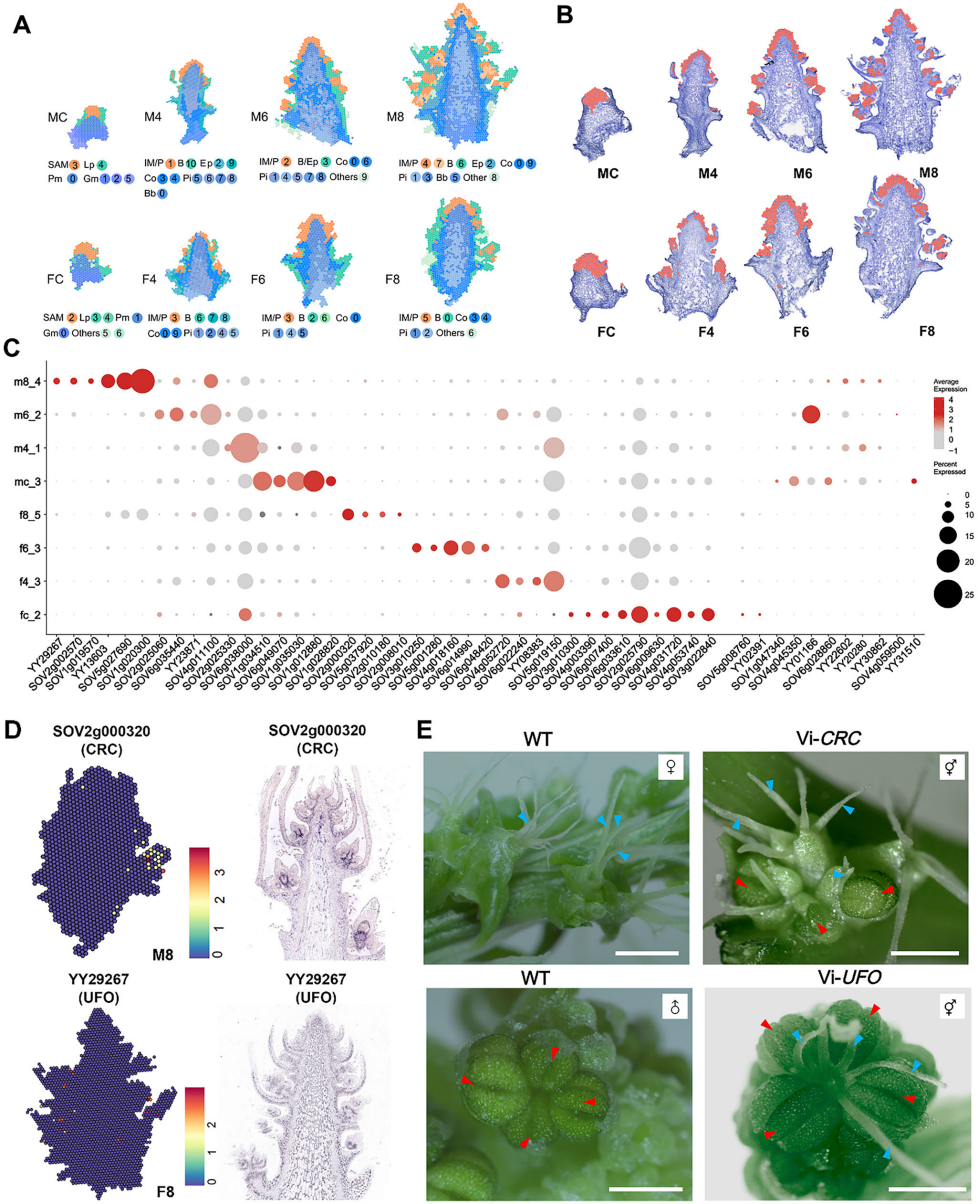
Spatiotemporal transcriptomic atlas reveals inflorescence development‐related clusters, stage‐specific marker genes, and functional validation of sex‐specific regulators in spinach. A) Clustering and annotation of functional cell populations based on ST data. SAM, shoot apical meristem; Lp, leaf primordium; Pm, pith rib meristem; Gm, ground meristem; IM/P, inflorescence meristem or flower primordia; B, bract; Ep, epidermis; Co, cortex; Pi, pith; Bb, basal bud; White scale bars, 1mm. B) Spatial distribution of inflorescence development‐related clusters. C) Representative stage‐specific marker genes identified in inflorescence development‐related clusters. Dot diameter indicates the proportion of cluster cells expressing a given gene. The color scale represents the gene expression levels, with red denoting high expression and gray corresponding to low expression. D) Validation of spatiotemporal expression patterns of *SpCRC* and *SpUFO*. The left pictures represent electronical gene expression using ST data; the right pictures show the experimental in situ hybridization at the 10‐leaf stage. E) Floral organ sex reversal in spinach following VIGS‐mediated knockdown of *SpCRC* and *SpUFO*. Blue arrow, stigma; Red arrow, stamen.

To trace dynamic spatiotemporal gene expression during early inflorescence development in spinach, we performed ST sequencing on developing shoot apices. Consistent with the structural analysis, shoot apex sections from both male and female plants at four developmental stages (MC, FC, M4, F4, M6, F6, M8, and F8) were subjected to ST sequencing using the BMKMANU platform. Notably, early spinach inflorescences are extremely small in size; even at the eight‐leaf stage, their length is only ≈1 mm (Figure 1B), which fits entirely within the 6.5 mm × 6.5 mm capture area of the S1000 chip. However, obtaining structurally intact frozen sections of these inflorescences posed significant technical challenges. Nevertheless, by optimizing the sectioning protocol and, critically, generating a large number of serial sections, we successfully obtained intact 10 µm thick frozen sections of both male and female inflorescences at all four developmental stages (**Figure** **2**B). These sections were mounted on four chips and used for ST sequencing (Figure S3, Supporting Information). The S1000 chip provides multi‐level resolution settings that can be finely tuned to suit different tissue types. Based on the cell size range of inflorescence tissues (8–20 µm) and a comparative analysis of ST data at different resolutions, the SupSpot level 4 setting (27 µm resolution) was determined to be optimal for this study (Figure S5A, Supporting Information). At this resolution, ≈20 000 unique genes were detected per sample. The number of SupSpots across the eight samples ranging from 237 to 2,086, with an average of 2,000 median genes detected per SupSpot (Table S1, Supporting Information). Gene expression patterns across the eight samples were analyzed using Uniform Manifold Approximation and Projection (UMAP) for dimensionality reduction and clustering. This analysis revealed distinct cell cluster distributions: MC (6), FC (7), M4 (11), F4 (10), M6 (10), F6 (6), M8 (10), and F8 (7) clusters (Figure S6, Supporting Information). Genes with significantly higher expression levels in specific clusters compared to all other cellular clusters were identified as cluster‐enriched marker genes. We analyzed the expression profiles of marker genes within distinct clusters to accurately associate each cluster with its corresponding cell types. Based on the anatomical information and the marker gene expression, these clusters were assigned to different functional populations (Figure 2A). In the MC sample, six clusters were mapped to four specific functional populations: clusters 0, 3, and 4 corresponded to the SAM, Lp, and Pith rib meristem (Pm), respectively, while clusters 1, 2, and 5 were collectively classified as ground meristem (Gm). Similarly, in the FC sample, seven clusters were mapped to the same four populations (SAM, Lp, Pm, and Gm), although clusters 5 and 6 remained unclassified. In the M4 sample, ten clusters were categorized into six functional populations: clusters 0, 1, and 10 corresponded to IM/P, B, and basal bud (Bb), respectively; clusters 2 and 9 contained epidermis (Ep) cells; clusters 3 and 4 were associated with Cortex (Co); and clusters 5, 6, 7, and 8 were collectively grouped as Pith (Pi). These distinct functional populations were also identified across the remaining five samples (Figure 2A). Given the study's focus on inflorescence development and sex differentiation, clusters annotated as IM and P were designated as “inflorescence development‐related clusters” for downstream analysis. These clusters were exhibited and extracted from each sample using Seurat (Figure 2B). The resulting data were subsequently merged and normalized to create a new expression matrix for further bioinformatic analyses (see Figure S7 (Supporting Information) for the analysis workflow).

To distinguish inflorescence development‐related clusters at different stages, we identified numerous stage‐specific marker genes through an analysis of the ST data. Developmental stage‐specific genes are those that are highly or uniquely expressed during one of the eight samples. Examples include *SpUBA2C* (SOV6g049070), *SpLNK2* (SOV1g012880) and *SpCKX7* (SOV1g034510) for MC; *SpFT* (SOV4g003390), *SpSAP* (SOV2g025790) and *SpANT* (SOV4g031720) for FC; *SpSRS5* (SOV2g025330) and *SpIMK2* (SOV6g038000) for M4; *SpBEL10* (SOV2g025060) and *SpCAL* (YY23871) for M6; *SpDDB1A* (SOV3g010250), *SpJMJ16* (SOV4g018160) and *SpDDL* (SOV6g014990) for F6; *SpUFO* (YY29267), *SpPI* (SOV2g002570), *SpSEP1* (SOV1g020300) and *SpAGL15* (SOV1g019570) for M8; *SpCRC* (SOV2g000320), *SpSPL13* (SOV5g037920) and *SpAIL5* (SOV2g010180) for F8 (Figure 2C; Table S3, Supporting Information). Among them, *SpUFO* encodes an F‐box protein that is exclusively expressed in male inflorescence and is implicated in the determination of male floral organ and floral meristem identity.^[^ ^26^, ^33^, ^34^, ^35^, ^36^ ^]^ In contrast, *SpCRC* encodes a YABBY transcription factor that is highly expressed in female inflorescence and plays a conserved role in gynoecium development.^[^ ^26^, ^37^, ^38^, ^39^, ^40^, ^41^ ^]^ Both genes were selected for validation of mRNA distribution and function via RNA in situ hybridization (ISH) and virus‐induced gene silencing (VIGS). The ISH results showed specific expression signals within their corresponding clusters, consistent with the ST data (Figure 2D). In the VIGS experiments, spinach plants carrying the *pTRV2* empty vector developed normal male or female flowers. However, in the female plants infected with the *pTRV2‐SpCRC* vector, *SpCRC* expression was significantly suppressed, resulting in disrupted floral organ development—some gynoecia were transformed into stamens. Conversely, in male plants with *SpUFO* knockdown, stamens were converted into gynoecia, accompanied by a marked reduction in *SpUFO* expression (Figure 2E). These results confirmed that *SpCRC* and *SpUFO* are key regulators of female and male floral organ formation, respectively, thereby supporting the reliability of our ST data.

In summary, we generated high‐quality spatiotemporal transcriptomic data using four S1000 chips, which revealed dynamic gene expression patterns during the development of male and female inflorescences. This enabled a deeper understanding of the molecular mechanisms underlying inflorescence development and sex differentiation.

### 2.3 Identification of Key Genes Involved in Floral Transition in Spinach

**Figure 3:**
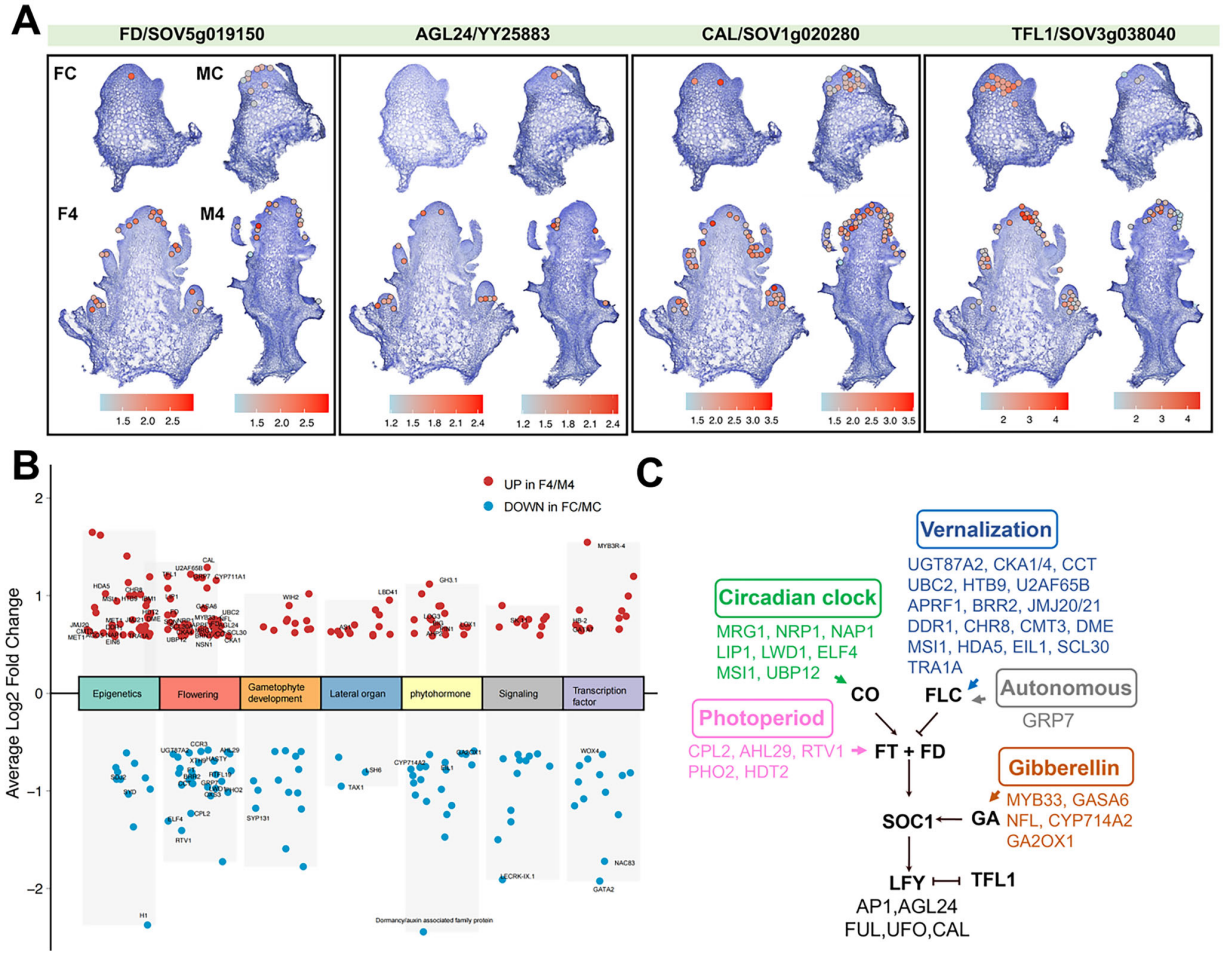
Key gene expression and regulatory network of floral transition in spinach. A) Spatiotemporal patterns of key flowering genes. B) GO enrichment analysis of DEGs between the cotyledon stage and the four‐leaf stage. C) The proposed gene regulatory network governing floral transition in spinach. Core flowering genes are shown in bold black, with genes associated with distinct flowering regulatory pathways indicated by different colors.

Spinach, a long‐day overwintering plant, requires coordinated regulation of photoperiod, vernalization, and other flowering‐related pathways to initiate floral transition. To elucidate the underlying molecular mechanisms, we performed a comparative analysis of DEGs between the F4 and FC samples, as well as between the M4 and MC samples, identifying 942 and 455 DEGs, respectively (Figure S8, Supporting Information). These DEGs are mainly involved in a variety of biological processes, including epigenetic regulation, flowering, lateral organ and gametophyte development, phytohormone metabolism, signaling, and transcription factors activity (**Figure** **3**B). By filtering DEGs functionally associated with floral transition based on gene annotations, we demonstrated that spinach shares conserved floral transition pathways found in other flowering plants: Vernalization pathway (*SpUGT87A2* (SOV6g026980), *SpUBC2* (SOV5g008040), *SpDME* (SOV5g021970), *SpU2AF65B* (SOV6g011220), *SpAPRF1* (SOV4g023690), *SpBRR2* (YY31594), *SpEIL1* (SOV2g006990), etc.), Photoperiod pathway (*SpRTV1* (SOV4g037130), *SpMRG1* (SOV3g043600), *SpUBP12* (SOV5g019210), *SpNRP1* (SOV6g043890), *SpLWD1* (SOV2g009880), *SpELF4* (SOV1g028600), *SpCPL2* (SOV3g036090), *SpAHL29* (SOV5g038710), *SpPHO2* (SOV6g010030), *SpMSI1* (SOV4g051130), etc.), Autonomous pathway (*SpGRP7* (YY01167)) and Gibberellin pathway (*SpGA2OX1* (SOV5g025800), *SpGASA6* (SOV5g008600), *SpCYP714A2* (SOV1g014760), *SpMYB33* (SOV5g023520), etc.) (Figure 3C; Table S6, Supporting Information). Among these, some key floral transition‐related genes, including *SpFD* (SOV5g019150), *SpAGL24* (YY25883), *SpCAL* (SOV1g020280), and *SpTFL1* (SOV3g038040) were further visualized using the ST data superimposed on tissue samples (Figure 3A). These findings provide additional evidence that the mechanisms underlying floral induction are highly conserved across flowering plants. The identification of these key regulatory genes offers a molecular framework for understanding the flowering regulatory network in spinach.

### 2.4 Pseudotime Trajectory Reveals Epigenetic Control of Sex Differentiation in Spinach

**Figure 4:**
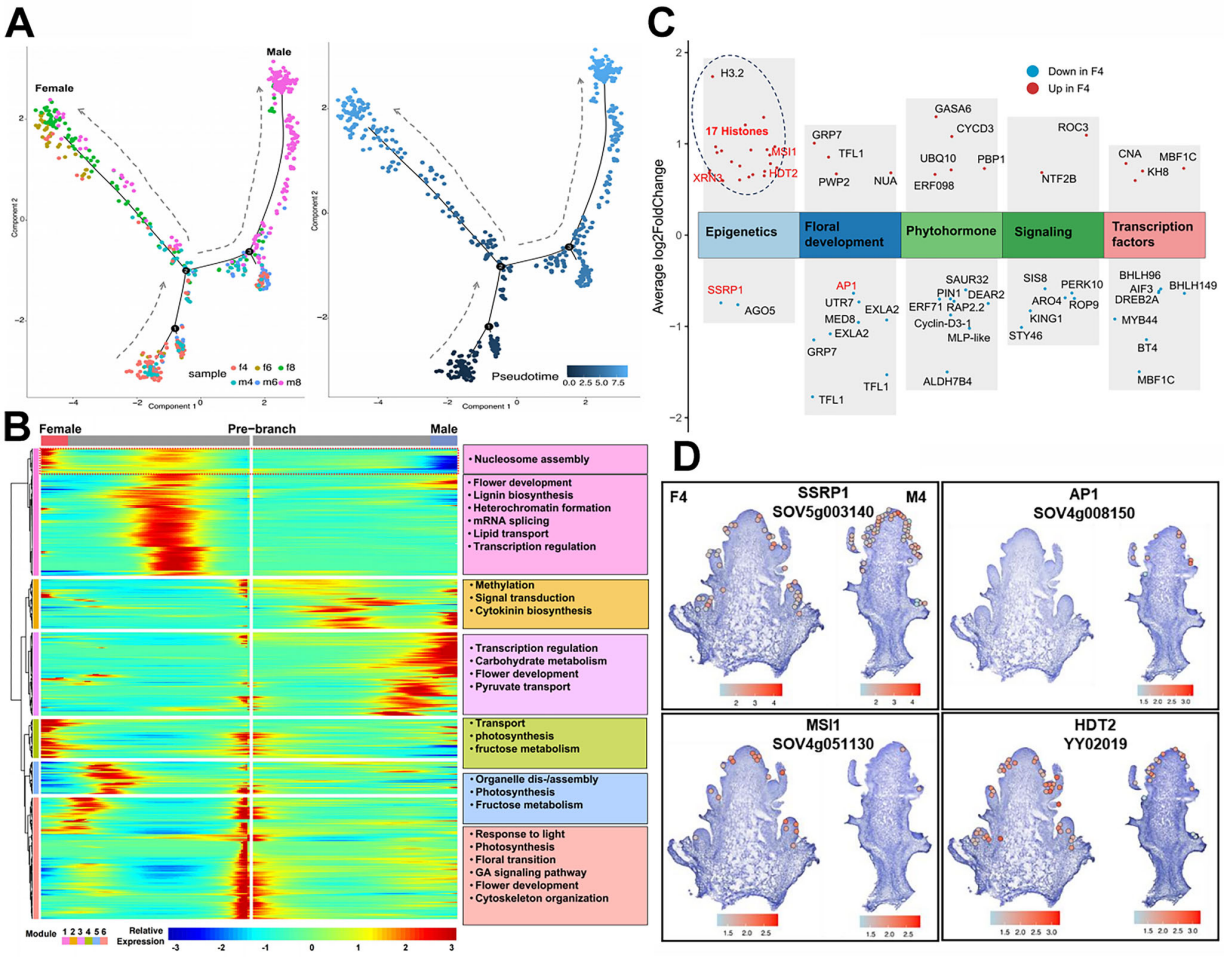
Unveiling the sex differentiation mechanisms by pseudotime trajectory and DEGs analysis. A) Pseudotime trajectory analysis of inflorescence development‐related clusters across four‐ to eight‐leaf stages reveals bifurcating developmental paths at the four‐leaf stage, marking the onset of sex differentiation, with *SpUFO* and *SpCRC* identifying male and female trajectories, respectively. B) The heatmap of six gene expression modules along pseudotime reveals distinct lineage‐specific transcriptional programs during sex differentiation. Genes significantly associated with branch point 2 are hierarchically clustered, with pseudotime progressing from the center of the heatmap outward, where the right side represents the male trajectory and the left side represents the female trajectory. C) GO enrichment analysis of DEGs between male and female inflorescences at the four‐leaf stage reveals several significantly enriched biological processes. D) Spatial expression patterns of key sex‐differentiation genes, such as *SpMSI1*, *SpHDT2*, and *SpSSRP1*, demonstrate sex‐specific expression at the four‐leaf stage, supporting their roles in regulating sex‐specific floral development via epigenetic mechanisms.

While spinach serves as an excellent model for investigating sex differentiation, the precise timing and underlying molecular mechanisms of this process remain poorly understood. To address these questions, we constructed a pseudotime trajectory of developing male and female inflorescences. This analysis revealed two distinct differentiation pathways at branch 2: one corresponding to male inflorescence differentiation, marked by *SpUFO*, and the other to female inflorescence differentiation, marked by *SpCRC* (**Figure** **4**A). Branch 2 is predominantly characterized by the presence of spots from M4 and F4, indicating that sex differentiation in spinach occurs at the four‐leaf stage (Figure 4A). A total of 631 genes at branch 2 were grouped into six expression modules, each enriched in specific biological processes (Figure 4B). Notably, some genes in module 1 exhibit expression trends that correlated well with the direction of sex differentiation, and were primarily involved in nucleosome assembly, suggesting that epigenetic regulation plays a key role in spinach sex differentiation (Figure 4B).

Consistently, GO enrichment analysis of 553 DEGs between male and female inflorescences at the four‐leaf stage further supports the involvement of epigenetic mechanisms in sex differentiation (Figure 4C). For example, the female inflorescence notably exhibited higher expression of histone variants, such as *SpH2A* (SOV6g029740) and *SpH2B.1* (SOV5g004250) (Figure 4C). Moreover, key epigenetic regulators including *SpMSI1* (SOV4g051130) and *SpHDT2* (YY02019) were highly expressed in the F4 stage, whereas *SpSSRP1* (SOV5g003140) showed higher expression in M4 (Figure 4D). Considering that MSI1 is a conserved subunit of both the Chromatin Assembly Factor (CAF‐1) and Polycomb Repressive Complex 2 (PRC2),^[^ ^42^, ^43^, ^44^, ^45^, ^46^ ^]^ and that SSRP1 is part of the Facilitates Chromatin Transcription (FACT) complex,^[^ ^47^, ^48^, ^49^, ^50^, ^51^ ^]^ it is plausible that these proteins act as molecular switches regulating sex differentiation in spinach.

Taken together, our results demonstrate that sex differentiation in spinach occurs at the four‐leaf stage and is tightly regulated by epigenetic mechanisms.

### 2.5 The SpMSI1‐SpHDT2 Complex is a Key regulator of Female Floral Differentiation in Spinach

**Figure 5:**
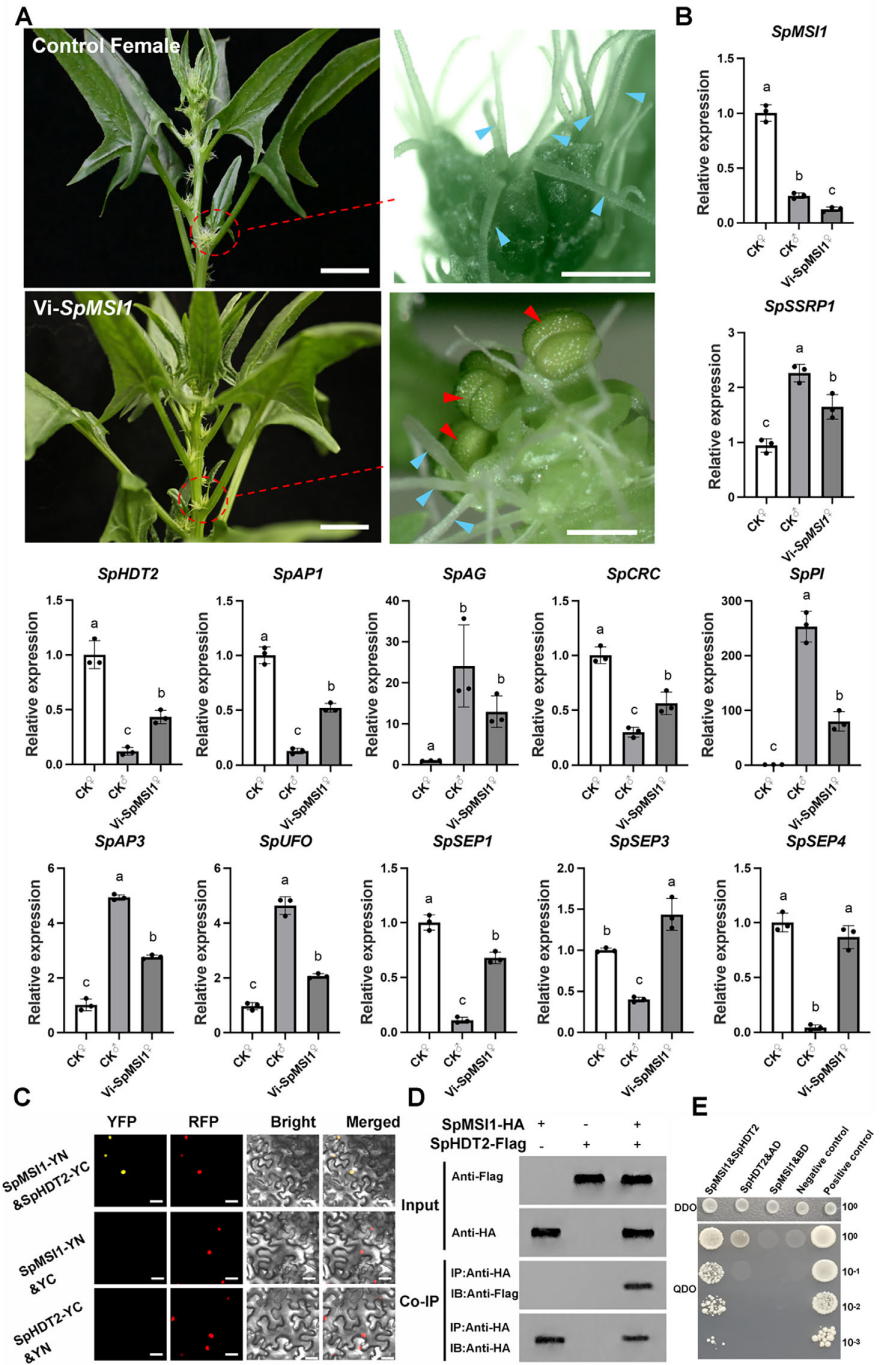
Functional validation of SpMSI1 in regulating female floral development in spinach. A) Phenotypic observations of VIGS‐mediated silencing of *SpMSI1* in female plants. Knockdown results in partial conversion of gynoecia into stamens, indicating disrupted female floral identity. Blue arrow: stigma; red arrow: stamen. Scale bars, 1 mm. B) Relative expression levels of floral organ identity genes in *SpMSI1*‐silenced plants assessed by qRT‐PCR. Silencing of *SpMSI1* resulted in up‐regulation of *SpSSRP1*, B‐class genes (*SpAP3*, *SpPI*, *SpUFO*), the C‐class gene (*SpAG*), and the E‐class gene (*SpSEP3*), and down‐regulation of *SpHDT2*, the A‐class gene (*SpAP1*), the C‐class gene (*SpCRC*), and the E‐class gene (*SpSEP1*). The expression of *SpSEP4* remained unaffected. These results suggest that *SpMSI1* regulates floral identity primarily by modulating multiple ABCE‐class genes. C–E) Protein‐protein interaction assays confirming the physical interaction between SpMSI1 and SpHDT2 using BiFC, Co‐IP, and Y2H analyses, respectively. In panel C), the red fluorescence marks nuclear localization, indicating that the SpMSI1‐SpHDT2 complex functions within the nucleus. Scale bars, 25 µm.

Our prior analysis suggested that the epigenetic regulator SpMSI1 plays a critical role in spinach sex differentiation by promoting female floral development. To investigate its genetic function, we used the VIGS to knock down *SpMSI1* expression in female spinach plants. Control plants harboring the empty *pTRV2* vector exhibited normal female floral development, whereas *SpMSI1*‐silenced plants produced some male flowers with four functional stamens producing viable pollen adjacent to pistils (**Figure** **5**A). These results demonstrate that *SpMSI1* is essential for maintaining female floral identity and repressing male floral formation.

To further elucidate the mechanism by which *SpMSI1* regulates sex differentiation, we analyzed the expression levels of 12 floral development‐related genes in *SpMSI1*‐silenced plants using qRT‐PCR (Figure 5B). In control plants, *SpSSRP1* expression was significantly higher in male flowers than in female flowers. Upon *SpMSI1* silencing, *SpSSRP1* was notably upregulated in female flowers, indicating that SpMSI1 may act as a negative regulator of *SpSSRP1*. Furthermore, SpMSI1 modulates the expression of A‐, B‐, C‐, and E‐ class floral identity genes. It positively regulates *SpAP1*, *SpCRC*, and *SpSEP1*, while negatively regulating *SpAG*, *SpPI*, *SpAP3*, *SpUFO*, and *SpSEP3*. Expression of *SpSEP4* remained unchanged following *SpMSI1* silencing. These findings indicate an antagonistic regulatory relationship between *SpMSI1* and *SpSSRP1*, wherein *SpMSI1* promotes female floral differentiation by enhancing C‐class genes expression and suppresses male flower formation by repressing B‐class genes expression.

Mechanistically, MSI1 functions in concert with histone‐modifying enzymes such as histone deacetylases to regulate plant development.^[^ ^42^, ^52^ ^]^ Interestingly, our ST data revealed that *SpMSI1* and *SpHDT2* are co‐expressed at high levels during the four‐leaf stage in female plants. Notably, *SpMSI1* silencing resulted in a significant down‐regulation of *SpHDT2*, suggesting that these two proteins may act as a complex. To test this hypothesis, we conducted Y2H, BiFC, and Co‐IP assays. These experiments confirmed a direct interaction between SpMSI1 and SpHDT2, with BiFC further localizing the complex to the nucleus (Figure 5C–E).

Our results indicate that during the critical four‐leaf stage in female plants, *SpMSI1* and *SpHDT2*, both involved in histone‐mediated repression, are upregulated. The SpMSI1‐SpHDT2 complex likely binds to the regulatory regions of floral development‐related genes or is recruited to these loci, where it decreases histone acetylation and increases H3K27me3 levels. This epigenetic repression complex suppresses the expression of flower development‐related genes, more likely B‐class genes, promoting female flower development and inhibiting male organ formation. In contrast, male plants exhibit lower expression of *SpMSI1* and *SpHDT2*, resulting in higher B‐class gene expression due to elevated histone acetylation and reduced H3K27me3 levels, thereby promoting male flower development and repressing female organogenesis (**Figure** **7**).

**Figure 6:**
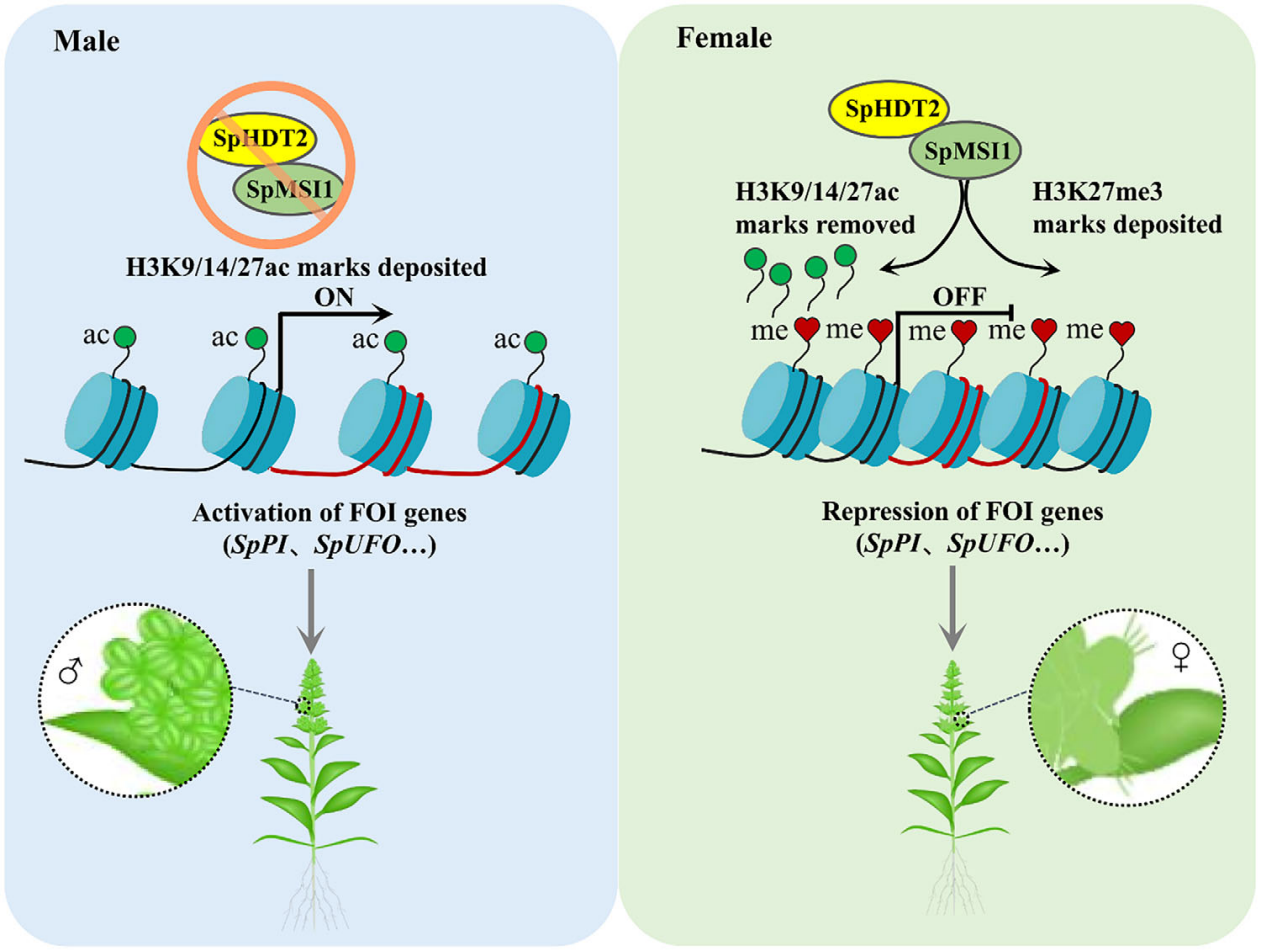
Proposed working model for the SpMSI1‐SpHDT2 complex's epigenetic regulation of sex differentiation in spinach. The sex differentiation in spinach is governed by an epigenetic mechanism centered on the SpMSI1‐SpHDT2 complex, which functions as a molecular switch at the four‐leaf stage, a critical phase for sex determination. In female plants, high expression of *SpMSI1* (a PRC2 component) and *SpHDT2* (a histone deacetylase) leads to removal of activating histone acetylation marks (H3K9/14/27ac) and deposition of repressive H3K27me3 marks, thereby silencing male‐promoting B‐class genes (*SpPI* and *SpUFO*) and activating female‐specific genes like *SpCRC*, culminating in gynoecium formation. In contrast, male plants exhibit low levels of *SpMSI1* and *SpHDT2*, may resulting in persistent activating histone marks and reduced repressive modifications, which activate B‐class genes and suppress female development, promoting stamen formation.

### 2.6 Dissecting the Floral Meristem and Organ Identity Gene Networks in Spinach

The floral transition culminates in the activation of floral meristem identity (FMI) genes, which trigger the formation of the floral meristems that ultimately give rise to flowers with multiple‐whorl floral organs. The spatial arrangement and identity of these organs are subsequently determined by floral organ identity (FOI) genes. To characterize these genetic components in spinach, we conducted a phylogenetic analysis to identify both FMI and FOI genes families. Our results revealed that spinach possesses all the conserved FMI genes previously reported in Arabidopsis, including LEAFY (LFY), SUPPRESSOR OF OVEREXPRESSION OF CO 1 (SOC1), APETALA1 (AP1), CAULIFLOWER (CAL), TERMINAL FLOWER1 (TFL1), AGAMOUS‐LIKE 24 (AGL24) and FRUITFULL (FUL) (Figure S9, Table S7, Supporting Information). Notably, some FMI genes, such as SpLFY and SpTFL1, have undergone gene duplication events (Table S7, Supporting Information), suggesting that spinach may have developed more sophisticated regulatory mechanisms governing floral meristem formation.

For FOI genes, we identified a total of 25 A‐, B‐, C‐, D‐, and E‐class genes. However, SEPALLATA2 (SEP2) is notably absent (Figure S9, Table S7, Supporting Information). Interestingly, spinach flowers lack petals entirely in both male and female forms (Figure S4, Supporting Information), raising the question of whether the absence of SpSEP2 is functionally linked to this morphological trait. Further investigation is needed to clarify this potential association. Moreover, we presented the differential spatiotemporal expression atlas of FMI and FOI genes, which support their presumed roles in floral development. For example, SpAP3 (SOV2g025350) and SpPI (SOV2g002570), both critical for stamen specification, are exclusively expressed during the M6 and M8 stages (**Figure** **6**).

**Figure 7:**
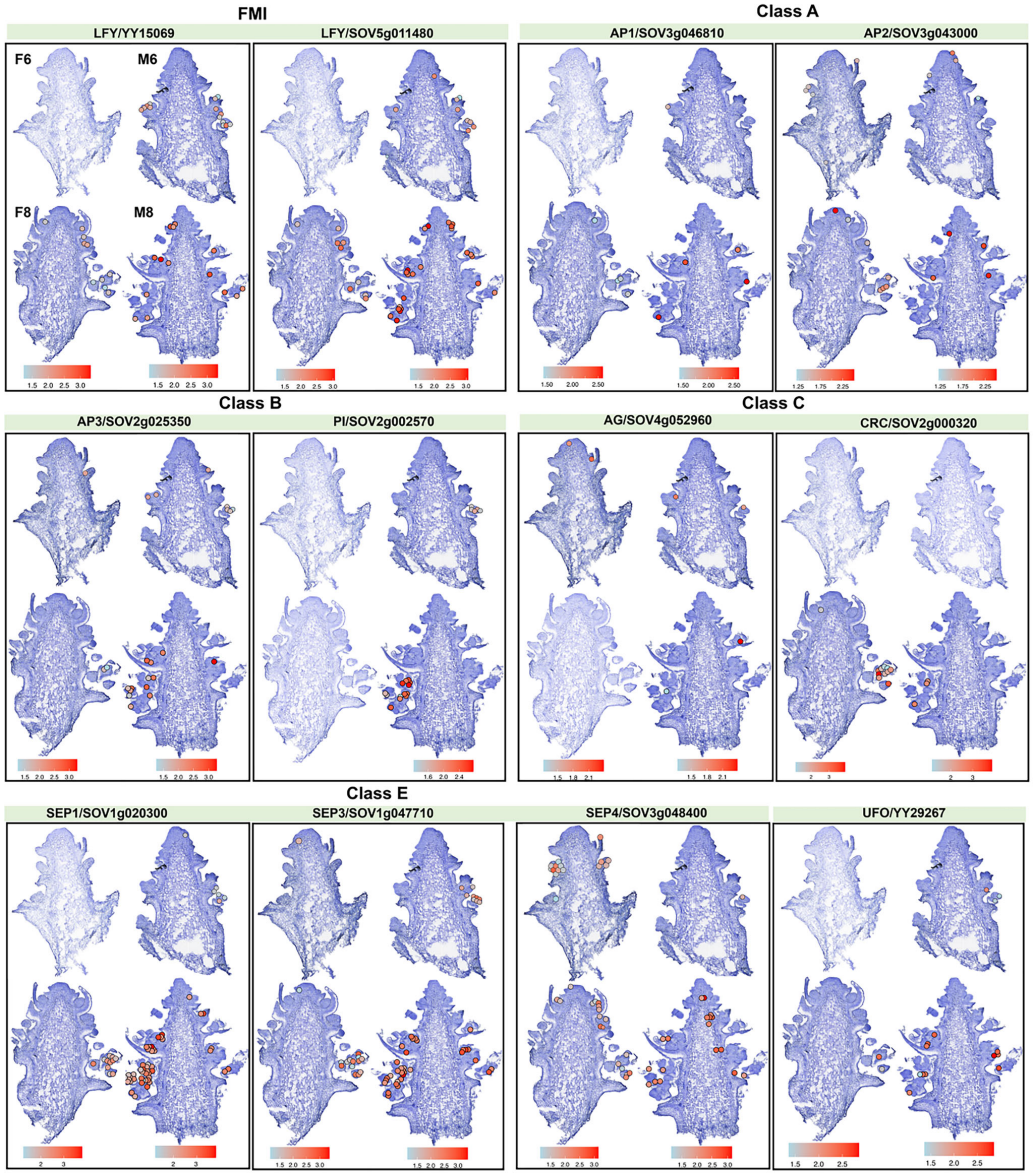
Spatiotemporal expression patterns of FMI and FOI genes in male and female inflorescences at the six‐ to eight‐leaf stage.

### 2.7 Complexity of Transcriptional Regulation and Phytohormone‐Mediated Signaling During Inflorescence Development

To gain a comprehensive understanding of the regulatory landscape underlying inflorescence development, we further explored the roles of transcription factors and phytohormone‐related genes. Transcription factors (TFs) and phytohormones play an important role throughout the entire process of inflorescence development. Using the ST data, we identified 1,119 TFs, which were categorized into 12 distinct clusters based on their expression patterns (Figure S10, Supporting Information). Among them, the number of TFs showing high expression at specific developmental stages was as follows: FC (177), F4 (143), F6 (169), F8 (142), MC (130), M4 (116), M6 (90), and M8 (152) (Table S8, Supporting Information). These findings suggest that specific TFs function at distinct stages of male and female inflorescence development.

In addition, we identified and functionally validated 379 phytohormone‐related genes (Table S9, Supporting Information). Notably, genes associated with peptide hormone and cytokinin (CK) biosynthesis were predominantly expressed in developing female inflorescences, while genes involved in brassinosteroid (BR) biosynthesis showed high expression in developing male inflorescences. Additionally, genes related to gibberellin (GA) biosynthesis exhibited a progressive increase in expression during the development of both male and female inflorescences (Figure S11, Supporting Information).

To further examine developmental characteristics in male and female inflorescences, we conducted a Weighted Gene Correlation Network analysis (WGCNA) of DEGs from MC to M8 and FC to F8, respectively. This analysis identified three distinct co‐expression modules in both male and female samples. GO functional annotations of these modules revealed enrichment in several biological processes, including carbohydrate metabolism, flower organ development, phytohormone signaling, epigenetic regulation, and transport (Figure S12, Supporting Information).

## 3 Discussion

Inflorescence development and sex differentiation are pivotal yet poorly understood processes in flowering plants. While transcriptome studies have identified some regulatory networks, they lack the spatial and temporal resolution necessary to fully capture gene expression dynamics during key developmental transitions.^[^ ^14^, ^53^, ^54^, ^55^, ^56^, ^57^ ^]^ In this study, we employed spatial transcriptomics to generate a genome‐wide expression atlas covering four critical developmental stages of male and female spinach inflorescences, from floral transition to organ formation. Spinach serves as an ideal model system due to its dioecious nature and small inflorescence size, which make it ideal for high‐resolution whole‐tissue spatial analyses.

Our data identified many stage‐ and sex‐specific marker genes, including SpUFO, SpCRC, and SpNRT1. SpUFO, encoding a conserved floral organ identity regulator, appears to cooperate with SpLFY to activate the SpPI or SpAP3 expression, suggesting a regulatory mechanism that warrants further functional validation in spinach. The conserved role of SpCRC in carpel development was supported by VIGS, which induced the homeotic transformation of carpels into stamens. Interestingly, SpNRT1, a Y‐linked nitrate transporter, displayed male‐specific expression from early developmental stages (MC‐M8), implicating a potential role for nutrient signaling in male inflorescence development.^[^ ^26^ ^]^ Remarkably, sex differentiation was found to occur as early as the four‐leaf stage—prior to the emergence of sepals—and coincided with divergent chromatin remodeling signatures between male and female plants. Whether these changes are regulated by sex chromosome‐specific mechanisms (X/Y‐linked) remains to be elucidated. A central finding of this study is the role of SpMSI1, a PRC2 component mediating H3K27me3 deposition. While MSI1 homologs regulate floral transitions and gametogenesis in plants,^[^ ^42^, ^45^, ^58^, ^59^, ^60^, ^61^, ^62^, ^63^, ^64^, ^65^, ^66^, ^67^, ^68^, ^69^ ^]^ our results suggest a novel role for SpMSI1 in spinach sex determination. Silencing SpMSI1 led to the partial conversion of female to male flowers, implicating it as a repressor of male floral programs during early carpel development. This finding aligns with observations in Arabidopsis, where AtMSI1 suppresses ectopic floral gene expression, pointing to both conserved and species‐specific roles in reproductive development.^[^ ^46^, ^52^, ^70^ ^]^ Furthermore, our study indicates that the SpMSI1‐SpHDT2 complex plays a crucial role in regulating spinach sex differentiation. As both components are involved in histone suppression,^[^ ^52^, ^67^ ^]^ we hypothesize that this complex represses flower development‐related genes, particularly B‐class genes, to promote female flower development while inhibiting male organs in female plants. Further research is needed to confirm this hypothesis by identifying target genes, examining chromatin state changes, and analyzing the function of these genes. Additionally, our study highlights the involvement of E‐class MADS‐box TFs in floral organ patterning. Notably, the absence of SEP2 in spinach is particularly intriguing and may be associated with the species’ complete lack of petals in both sexes — an evolutionary adaptation that deserves further molecular and evolutionary analysis. Hormonal profiling further revealed male‐biased expression of CK and peptide hormone biosynthesis‐related genes, while BR biosynthesis genes were predominantly expressed in female flowers. This expression pattern aligns with the known role of CK in promoting male development.^[^ ^71^, ^72^, ^73^ ^]^ Together, these findings provide a comprehensive spatiotemporal framework for understanding the complex regulatory landscape of inflorescence and sex differentiation in spinach.

## 4 Experimental Section

### Plant Materials and Growth Conditions

The spinach inbred line Sp75 was selected for this study due to its short life cycle in laboratory conditions and the relatively synchronized development of male and female inflorescences. The plants were cultivated in a greenhouse under a photoperiod of 16 h light/8 h dark at a temperature of 20 °C. Inflorescence samples from male and female plants at the cotyledon, four‐leaf, six‐leaf, and eight‐leaf stages were harvested ≈12, 17, 21, and 24 days after planting (see Figure S1, Supporting Information), respectively. T11A primers were used to determine the sex of the early seedlings.

### SEM Observation

Male and female inflorescence samples from the stem apex were dissected under a stereomicroscope. Tissues were immediately fixed in FAA fixative (50% ethanol, 5% glacial acetic acid, and 1.85% formaldehyde; v/v/v) in 1.5 mL centrifuge tubes, followed by vacuum infiltration for 10 min to facilitate fixative penetration. After 4 h, the fixative was replaced with fresh FAA, and samples were kept at 4 °C overnight. The Samples were then dehydrated through a graded ethanol series (50%, 70%, 80%, 90%, and three changes of 100%), with each step lasting 25 min. This was followed by dehydration in tert‐butanol for 20 min. Samples were rapidly frozen in liquid nitrogen for 5 min and freeze‐dried under vacuum for 2–3 h, with duration adjusted to ensure complete drying. Finally, gold sputter coating was performed at 10 mA for 20 s per cycle, with 1 min intervals between cycles, and repeated 2–3 times. The samples were examined using a scanning electron microscope (Hitachi TM3030 Plus) to trace inflorescence developmental stages and distinguish morphological differences among floral organs.

### Tissue Fixation, Staining and Imaging

Inflorescence samples were soaked in 75% OCT solution (Sakura) under vacuum for 10 min to ensure optimal infiltration and minimize air pockets. The samples were then embedded in 100% OCT. While still in the OCT medium, they were positioned under a stereomicroscope using fine dissection needles and carefully oriented along the apical‐basal axis to facilitate longitudinal sectioning. This orientation maximized the exposure of inflorescence structures. After embedding, the samples were snap‐frozen in isopentane pre‐chilled with liquid nitrogen and stored at −80 °C until further processing. The frozen blocks were mounted onto the specimen holder of a cryostat, and the angle between the tissue block and the blade was adjusted to ensure that the longitudinal plane of the tissue was parallel to the blade. Samples were cryosectioned longitudinally at −20 °C into 10 µm thick sections and transferred onto Baichuang S1000 gene expression chips (BMKGENE, China), which feature uniform capture areas of 6.8 × 6.8 mm, each containing 2 000 000 barcoded spots. For fixation and staining, the chips were incubated at 37 °C for 1 min, fixed in 3.8% formaldehyde in PBS for 30 min, and washed with PBS buffer. After washing, the chips were dehydrated with isopropanol and stained with 0.05% tolonium chloride (Merck). Finally, stained tissues were imaged using the Metafer Slide Scanning platform (MetaSystems, Germany).

### VIGS‐Based Gene Silencing in Spinach

VIGS was performed using the pTRV2 vector, which is derived from the tobacco rattle virus. ≈300 bp fragments from the coding sequences of target genes were amplified and cloned into the pTRV2 vector. Recombinant constructs were transformed into Agrobacterium tumefaciens strain GV3101 using the freeze‐thaw method (primer sequences are listed in Table S10, Supporting Information). Spinach seedlings at the first true leaf emergence stage (15 DAP) were mechanically inoculated with A. tumefaciens cultures carrying one of the following constructs: pTRV2‐empty (negative control), pTRV2‐SpPDS (positive control), pTRV2‐SpCRC, pTRV2‐SpUFO, pTRV2‐SpMSI1, or pTRV2‐SpSSRP1. Following inoculation, plants were maintained in darkness for 24 h and then transferred to standard growth conditions for recovery and gene silencing assessment. The experiments were performed in three biological replicates.

### RNA In Situ Hybridization

Antisense probes targeting SpCRC and SpUFO were designed using the Saiweier Signal Amplification Multiplex Isothermal (SweAMI) system and labeled with digoxigenin‐alkaline phosphatase (DIG‐AP). Primer sequences are listed in Table S10 (Supporting Information). Inflorescence samples were fixed in FAA solution (45% anhydrous ethanol, 6% acetic acid, and 5% formaldehyde) for more than 12 h, embedded in paraffin (Merck), and sectioned at a thickness of 10 µm. Sections were baked at 62 °C for 2 h, dewaxed in xylene, and pre‐treated with pre‐hybridization buffer (Servicebio). Hybridization was carried out overnight at 40 °C in a hybridization solution (10% formamide, 5× Denhardt's solution, 4 × SSC) containing the DIG‐labeled probes. Following hybridization, the sections were washed and stained with BCIP/NBT substrate solution (Merck). Images were acquired using a light microscope.

### qRT‐PCR Analysis

Total RNA was extracted from the inflorescence samples using the RNeasy Plant Mini Kit (Omega) and reverse‐transcribed into cDNA. qRT‐PCR was performed to quantify gene expression levels using the TB Green Premix FAST qPCR kit (Takara), with SpUBI as an internal control. The qRT‐PCR data were analyzed using the 2^‐ΔΔCt^ method (primers listed in Table S10, Supporting Information).^[^ ^74^ ^]^ Each qRT‐PCR assay was conducted using at least three independent biological samples, and three technical replicates for each sample.

### Identification and Phylogenetic Analysis of FOI and FMI Genes

To identify potential FOI and FMI genes in spinach, BLAST searches were performed against the spinach genome using Arabidopsis thaliana homologs as queries. Protein sequences of the resulting candidate genes were manually curated to confirm the presence and integrity of conserved domain architectures. A maximum‐likelihood phylogenetic tree was constructed using IQ‐TREE (v2.2.5; http://www.iqtree.org), incorporating spinach candidate genes along with known Arabidopsis genes involved in floral organ patterning and meristem identity.

### Library Construction and Sequencing

The optimal permeabilization time for fresh‐frozen tissues was determined to be 6 min, enabling maximal mRNA release, which was subsequently captured on spatially barcoded spots. cDNA was synthesized from the captured mRNA and used for sequencing library preparation following the manufacturer's protocol for the Gene Expression Kit (BMKMANU S1000). Sequencing was performed on the Illumina NovaSeq platform. The spinach reference genome used in this study was assembled by integrating autosomal and X chromosome sequences from the Monoe‐Viroflay cultivar (http://spinachbase.org/ftp/genome/Monoe‐Viroflay/) with the Y chromosome of Cornell‐NO.9 (BioProject PRJCA004899; https://ngdc.cncb.ac.cn/bioproject/browse/PRJCA004899).^[^ ^26^, ^75^ ^]^ Raw sequencing reads were filtered using the fastp software^[^ ^76^ ^]^ with parameters “‐Q ‐y ‐g ‐Y 10 ‐l 60 ‐b 150 ‐B 150,” and the obtained clean data were aligned to the combined reference genome using STAR aligner (v2.4.1d) with default parameters.^[^ ^77^ ^]^ For downstream analysis, tissue‐covered spots with optimal resolution were selected and visualized using BSTViewer (v1.42).

### Unsupervised Clustering and Cluster‐Enriched Marker Gene Identification

Gene expression matrices for each sample were processed for quality control and normalization using the Seurat R package (v4.1.0).^[^ ^78^ ^]^ Scaled data were subjected to principal component analysis (PCA) for dimensionality reduction, followed by Uniform Manifold Approximation and Projection (UMAP) for clustering. The clustering was performed using 3000 highly variable genes, 25 principal components, and resolution parameters (ranging from 1.7 to 2.1). Cluster‐enriched genes were identified via the FindAllMarkers function in Seurat,^[^ ^79^ ^]^ applying thresholds of |log_2_FC| > 0.25 and adjusted *p*‐value <0.01.

### Expression Matrix Construction and DEG Identification

Inflorescence development‐related clusters were defined based on marker genes and histological information from stained sections. The following clusters were selected: FC_cluster 2, F4_cluster 3, F6_cluster 3, F8_cluster 5, MC_cluster 3, M4_cluster 1, M6_cluster 2, and M8_cluster 4. These clusters were merged using the Seurat package to generate a combined dataset. The merged data were normalized, and a new expression matrix in RDS format was generated for downstream analyses. The stage‐specific genes were identified by comparing gene expression levels among the clusters, with expression levels >0.4 and expressed in >50% of cells within a cluster. DEGs were identified by comparing gene expression levels among the clusters, with |log_2_FC| > 0.58 and a *p*‐value <0.05.

### Construction of Sex Differentiation Pseudotime Trajectories

Pseudotime trajectories of inflorescence development‐related clusters across different developmental stages were constructed using the DDRTree algorithm implemented in Monocle2 (v2.18.0).^[^ ^21^ ^]^

Branch‐dependent gene expression analysis was performed using BEAM (Branch Expression Analysis Modeling) to identify genes associated with branch point 2, with a significance threshold of *p*‐value <0.05. Expression patterns of these branch‐specific genes were visualized as heat maps. Branch point 2 was chosen to inspect and the heatmap shows changes in two lineages at the same time. Columns were points in pseudotime, and rows correspond to individual genes. The center of the heatmap marks the start of the trajectory (referred to as the pre‐branch state). Genes were hierarchically clustered to form modules exhibiting similar lineage‐specific expression patterns. Subsequently, cluster‐specific gene sets were analyzed for GO enrichment using the ClusterProfiler R package (v4.0) to identify significantly enriched biological processes.

### Weighted Gene Co‐Expression Network Analysis

WGCNA was conducted using the WGCNA R package (v1.71). A dataset consisting of the 3000 highly variable genes (HVGs) derived from inflorescence development‐related clusters was used as input. A soft‐threshold power of 8 and a minimum module size of 80 genes were selected to construct a scale‐free network and detect co‐expression modules. Gene connectivity scores were calculated for each module. The resulting modules underwent GO enrichment analysis using the ClusterProfiler package (v4.6.0) to identify significantly enriched biological processes within each module.

### Time Series Expression Pattern Analysis

Gene sets encoding transcription factors and phytohormone‐related genes in spinach were retrieved from PlantTFDB (https://planttfdb.gao‐lab.org) and PHGD (http://phgd.bio2db.com), respectively. After manual curation, these gene sets were subjected to Time Series Expression Pattern analysis using the Mfuzz R package (V1.71). Genes were clustered based on their temporal expression profiles to identify stage‐specific expression patterns across inflorescence development.

### Yeast Two‐Hybrid Assay

To validate the protein interaction, coding sequences of SpMSI1 and SpHDT2 were cloned into pGADT7 and pGBKT7 vectors, respectively by double‐enzyme digestion and ligation. The resulting recombinant plasmids were co‐transformed into Saccharomyces cerevisiae strain AH109 using the lithium acetate method.^[^ ^80^ ^]^ Transformed yeast cells were plated on SD/‐Leu/‐Trp (‐LW) dropout medium for selection and subsequently transferred to SD/‐Leu/‐Trp/‐His/‐Ade (‐L‐W‐H‐A) medium to assess protein‐protein interactions. Serial dilutions of yeast cultures were spotted onto the media and incubated at 30 °C for 3 days. Controls were pGADT7‐T + pGBKT7‐53 (positive control) and pGADT7‐T + pGBKT7‐lam (negative control). Primer sequences used for cloning are listed in Table S10 (Supporting Information). The experiment was performed in two independent biological replicates.

### Bimolecular Fluorescence Complementation Assay

The coding sequences of SpMSI1 and SpHDT2 were cloned into the pEarleyGate201‐HA (containing the N‐terminal fragment of YFP, YFP^N^) and pEarleyGate202‐Flag (containing the C‐terminal fragment of YFP, YFP^C^), respectively, to generate the recombinant constructs SpMSI1‐YFP^N^ and SpHDT2‐YFP^C^. A nuclear localization marker plasmid was co‐transformed as a subcellular reference. All constructs were introduced into Agrobacterium tumefaciens strain GV3101. Agrobacterium cultures were infiltrated into Nicotiana benthamiana leaves using a needleless syringe, infiltrated plants were grown under long‐day conditions (16 h light, 8 h dark) at 22 °C. Infiltrated leaves were harvested 72 h post‐infiltration, and fluorescence signals were observed using a confocal laser scanning microscope (Leica TCS SP8, Germany). The experiment was performed in two independent biological replicates.

### CO‐Immunoprecipitation Assays

The coding sequences of SpMSI1 and SpHDT2 were cloned into the pEarleyGate201‐HA and pEarleyGate202‐Flag vectors, respectively, to generate the recombinant constructs SpMSI1‐HA and SpHDT2‐Flag. These plasmids were co‐expressed in Nicotiana benthamiana leaves via A. tumefaciens‐mediated infiltration. Control combinations included SpMSI1‐HA with an empty vector and SpHDT2‐Flag with an empty vector. For Co‐IP, total protein extracts were prepared from infiltrated leaves and incubated with anti‐HA magnetic beads (Merck) at 4 °C for 3 h. The beads were washed four times with lysis buffer (25 mM Tris‐HCl pH 7.4, 75 mm NaCl, 0.1% NP‐40), and the bound protein complexes were eluted and denatured in SDS loading buffer at 95 °C for 10 min. Both input and immunoprecipitated proteins were separated on 10% SDS‐PAGE gels, transferred onto PVDF membranes (Merck), and probed with anti‐Flag (1:2000; Abcam) and anti‐HA‐peroxidase (1:5000; Abcam) antibodies. Chemiluminescent signals were visualized using an ECL detection system (Bio‐Rad). The experiment was performed in two independent biological replicates.

### Statistical Analysis

All statistical analyses were performed using R software (v4.2.1) and GraphPad Prism (v9.0). For ST data, DEGs were identified using the Wilcoxon rank‐sum test with Bonferroni‐adjusted *p*‐values <0.05 and |log₂ fold change| >0.58 considered significant. For qRT‐PCR experiments, data are presented as mean ± standard deviation (SD) from three independent biological replicates. Prior to statistical testing, data were assessed for normality using the Shapiro‐Wilk test and for homogeneity of variance using Levene's test. Statistical comparisons between groups were then conducted using unpaired two‐tailed Student's t‐tests. A *p*‐value <0.05 was considered statistically significant.

## Conflict of Interest

The authors declare no conflict of interest.

## Author Contributions

C.Y. and H.Y. contributed equally to this work. The study was conceptualized by H.Y., W.J.G., and S.F.L., with methodology designed by C.Y., H.Y., Y.Y.Z., X.N.W., and Y.L.Z.; data visualization was carried out by C.Y., H.Y., S.J.W., and N.C., while supervision was provided by W.J.G. and S.F.L.; the original draft was written by C.Y. and H.Y., and subsequently reviewed and edited by H.Y., W.J.G., S.F.L., L.X.L., and W.Q.

## Supporting information



Supporting Information

Supplemental Tables

## Data Availability

All data are available in the main text or the supplementary materials. The raw spatial transcriptomic sequencing data have been deposited in the National Genomics Data Center (NGDC) under accession code (CRA024715) and are accessible at https://ngdc.cncb.ac.cn/gsa/s/085RKzU7.
