# Supplementary material for: Spatiotemporal Transcriptomic Atlas Reveals the Regulatory Mechanisms Underlying Early Inflorescence Development and Sex Differentiation in Spinach
Source: Adv Sci (Weinh). 2025 Jun 26;12(36):e07818. doi: 10.1002/advs.202507818 (PMC12462940; doi:10.1002/advs.202507818)
Supplement: Supplementary file 1 — Supporting Information [file ADVS-12-e07818-s001.docx]

Supporting Information

Spatiotemporal Transcriptomic Atlas Reveals the Regulatory Mechanisms Underlying Early Inflorescence Development and Sex Differentiation in Spinach

Chen You, Hao Yang*, Yueyan Zhao, Xiaoning Wang, Shuaijie Wei, Ning Chen, Yulan Zhang, Luxian Liu, Wei Qian, Shufen Li*, Wujun Gao*

Figs. S1 to S12


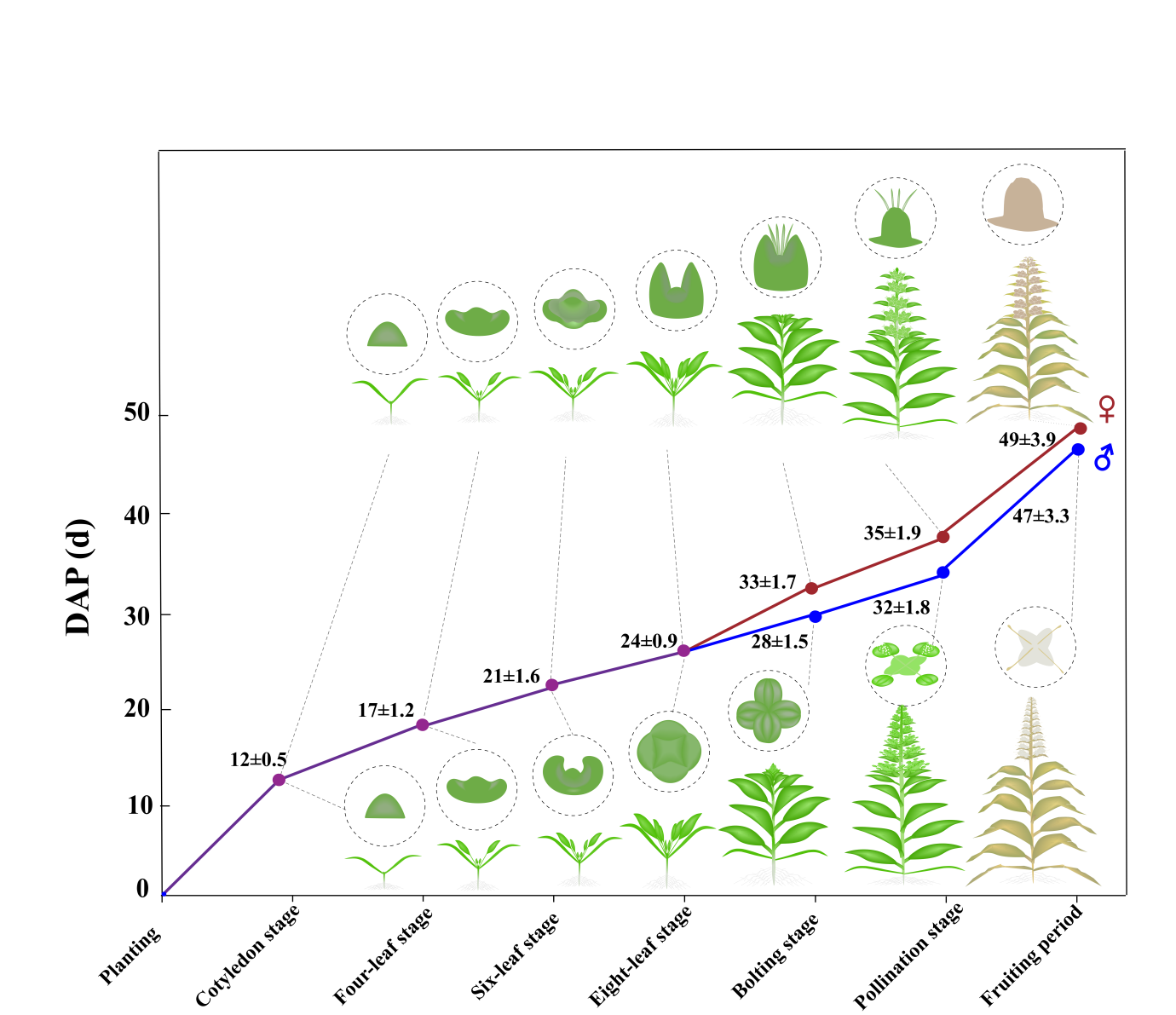


**Figure S1.** Schematic diagram of the spinach growth cycle under laboratory conditions.

Illustration of the growth and developmental stages of the spinach inbred line Sp75 cultivated under controlled conditions (16 h light / 8 h dark at 20 °C). DAP: days after planting.

**
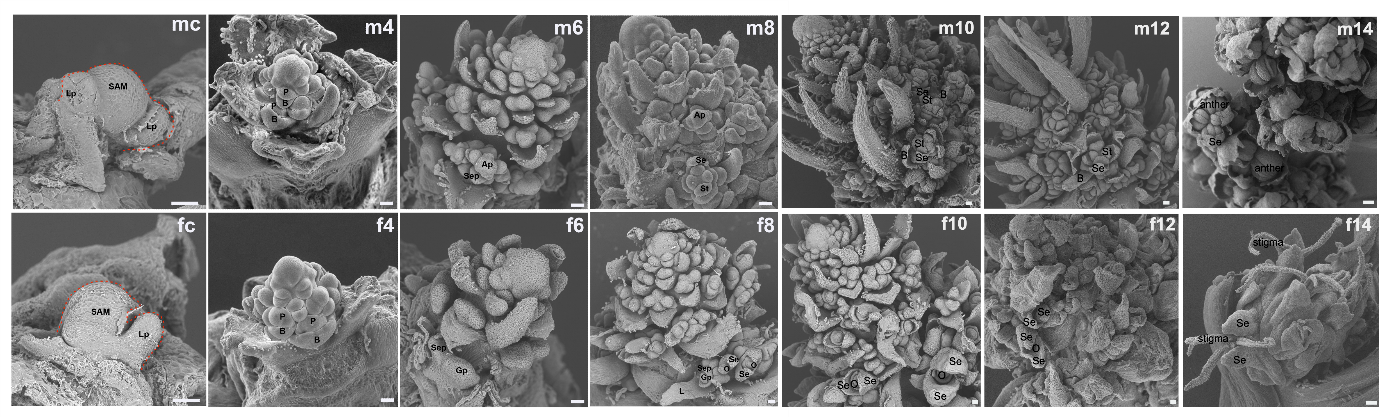
Figure S2.** Scanning electron microscopy of the developing male and female inflorescences. During these stages, the cotyledon stage, four-leaf, six-leaf, and eight-leaf stage represent key phases of early inflorescence development and were selected for spatiotemporal transcriptomic analysis. The 14-leaf stage corresponds to the bolting stage. SAM, shoot apical meristem; Lp, leaf primordium; IM, inflorescence meristem; P, flower primordia; B, bract; Stp, stamen primordia; Gp, gynoecium primordia; Sta, stamen; O, ovule; Sep, sepal. White scale bars, 100 μm.


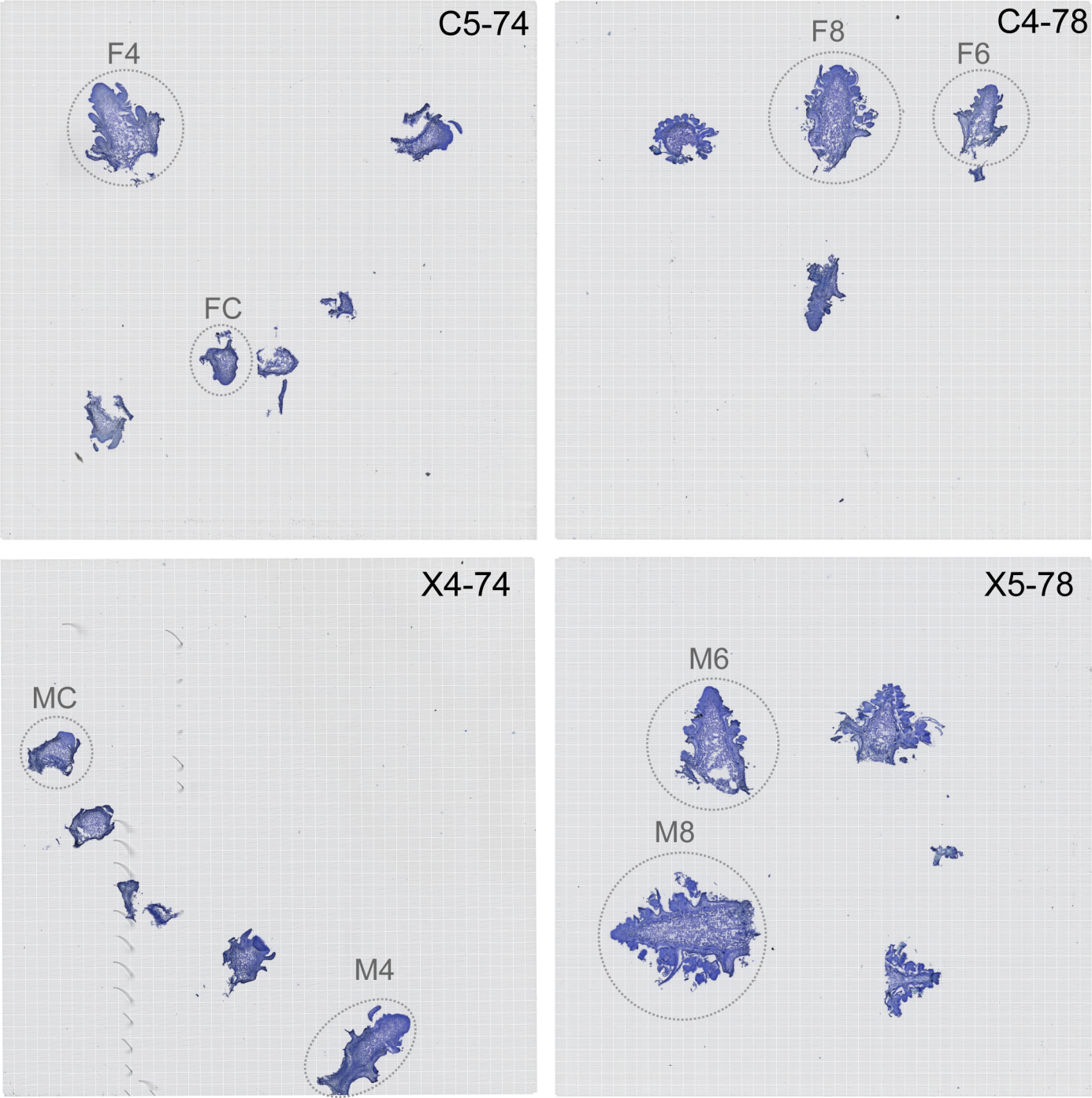


**Figure S3.** Longitudinal cryosections of male and female spinach inflorescences at four developmental stages: cotyledon (male cotyledon, MC; female cotyledon, FC), four-leaf (M4/F4), six-leaf (M6/F6), and eight-leaf (M8/F8). The cryosections were mounted on S1000 chips.


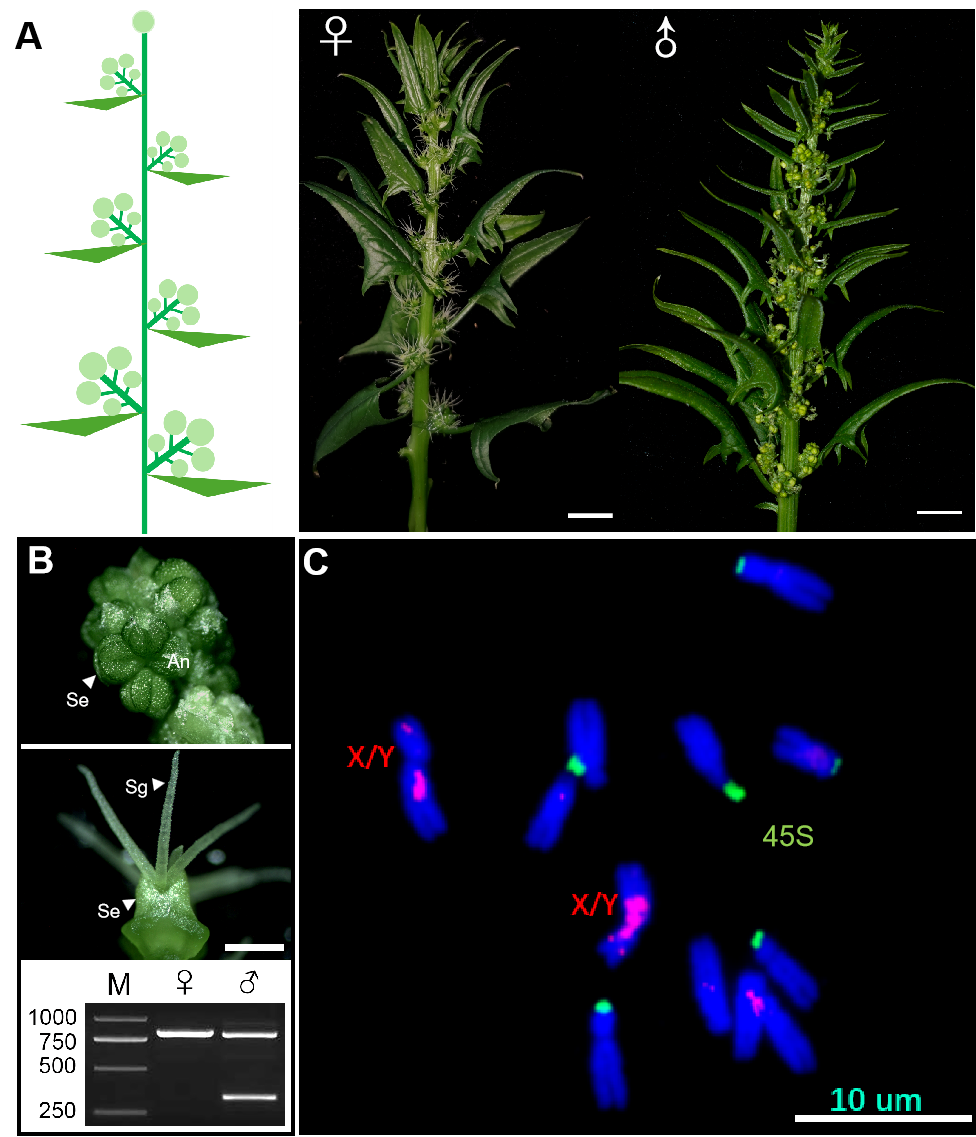


**Figure S4.** Morphology and sex identification of male and female spinach inflorescences. A) Morphology of male and female spinach inflorescences. Spinach exhibits a basal rosette architecture with a compound, thyrsus-like inflorescence. Dimorphic floral glomerules (male and female) develop in the axils of bracts and are arranged in a panicle-like pattern along the apical region of the stem. Female glomerules are positioned more proximally to the bracts compared to their male counterparts. B) PCR-based genotyping of juvenile spinach plants using sex-linked specific primers. Primers targeting the 5'-UTR of T11A produced a 321-bp diagnostic fragment specifically in male genotypes. An, anther; Sg, stigma; Se, sepal. C) FISH analysis of spinach autosomes and sex chromosomes at mitotic metaphase. Sex chromosomes were labeled using a probe derived from a single-copy sequence located at positions 19,985,411-19,996,798 bp on chromosome 4 of the genome assembly.

**Figure S5.** Spatial resolution selection and quality analysis of ST sequencing data. A) Comparison of ST sequencing data at different resolutions. B) Number of RNA molecules with unique molecular identifiers (UMIs) and the number of expressed genes detected per chip. The y-axis represents the number of transcripts per spot. C-D) Density distribution of UMIs and expressed genes across each sample.


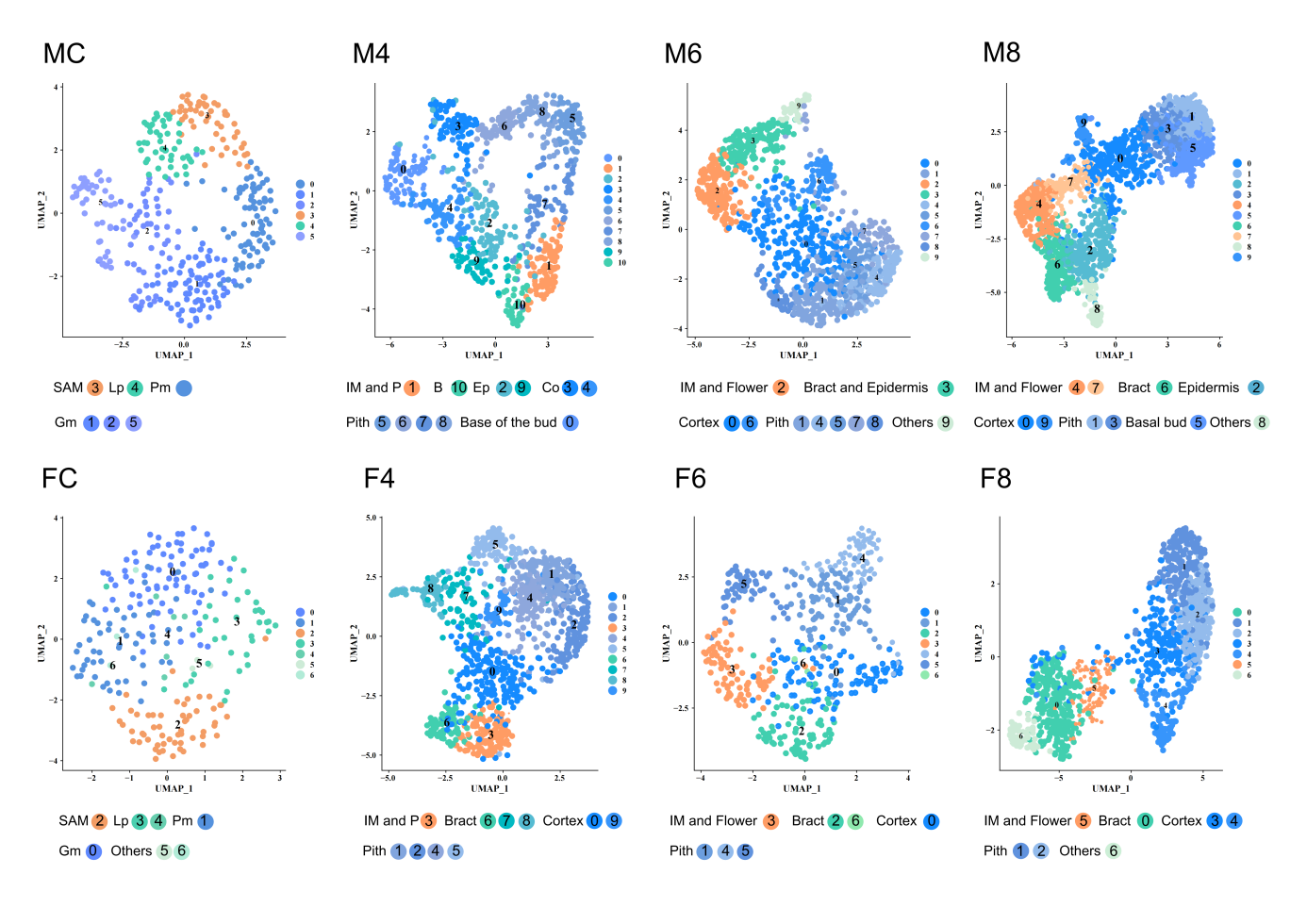


**Figure S6.** The UMAP unbiased clustering of the developing male and female inflorescence samples. Each dot represents an individual cell, with colors indicating the corresponding cell clusters. SAM, shoot apical meristem; Lp, leaf primordium; Pm, pith rib meristem; Gm, ground meristem; IM/P, inflorescence meristem or flower primordia; B, bract; Ep, epidermis; Co, cortex; Pi, pith; Bb, basal bud.


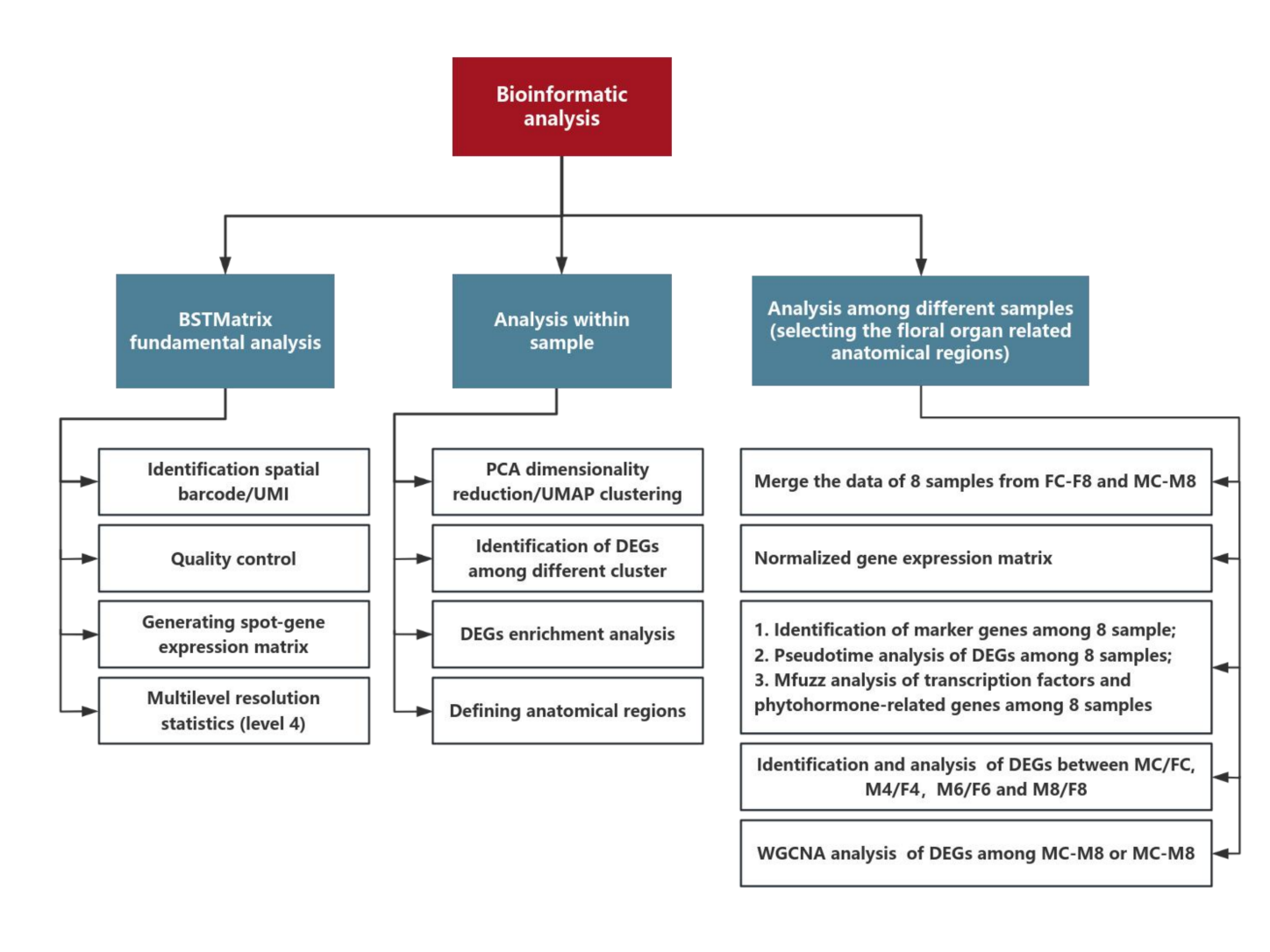


**Figure S7.** The workflow of bioinformatics analysis conducted in this study.


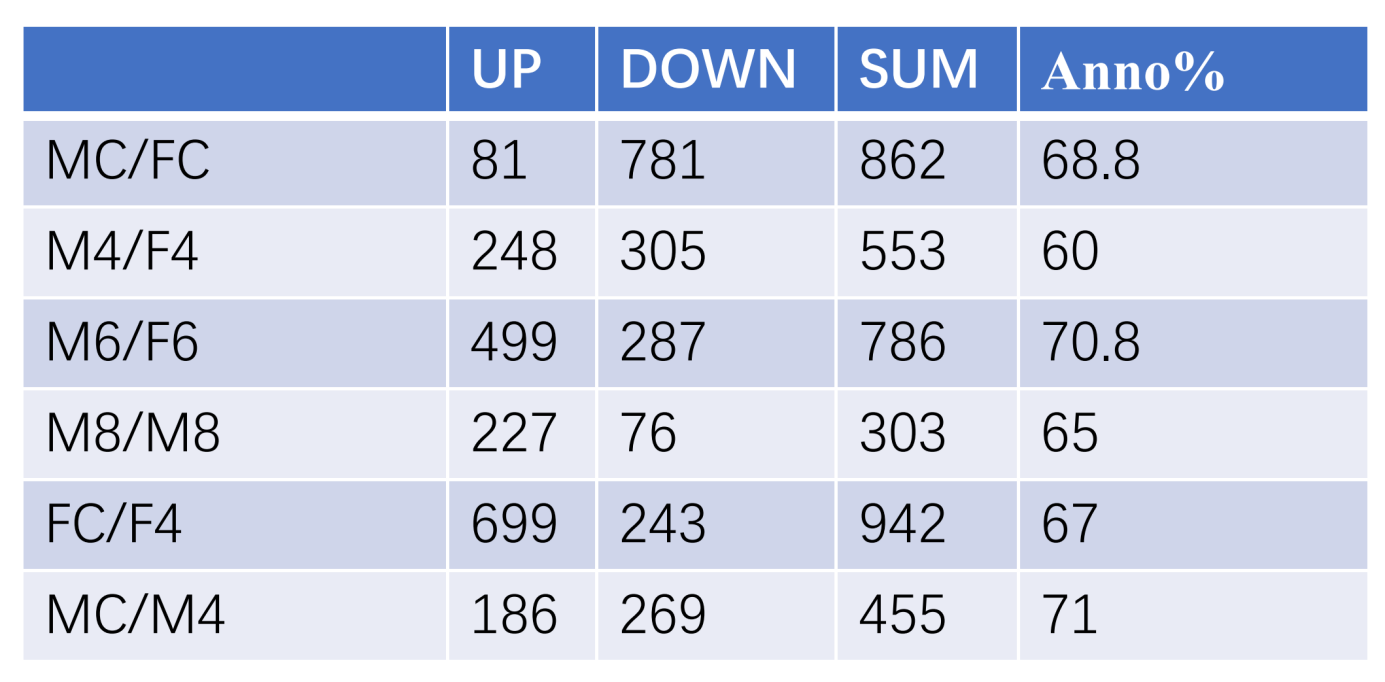


**Figure S8.** The number of DEGs among developing female and male inflorescence. Anno% indicates the functional annotation ratio of identified DEGs.


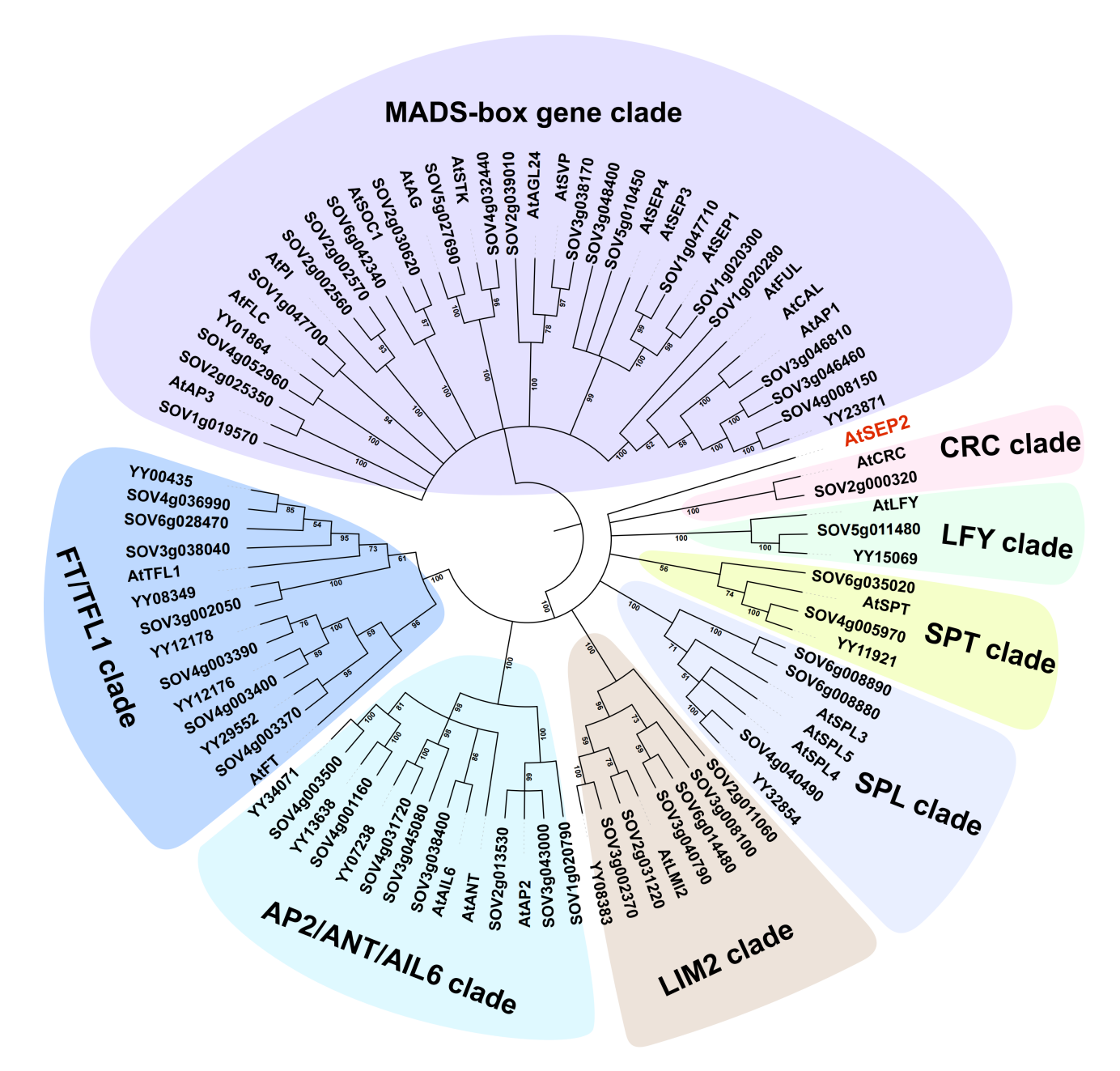


**Figure S9.** Phylogenetic analysis of floral meristem identity and floral organ identity genes in spinach. A maximum-likelihood phylogenetic tree was constructed using IQ-TREE v2.2.5.

**
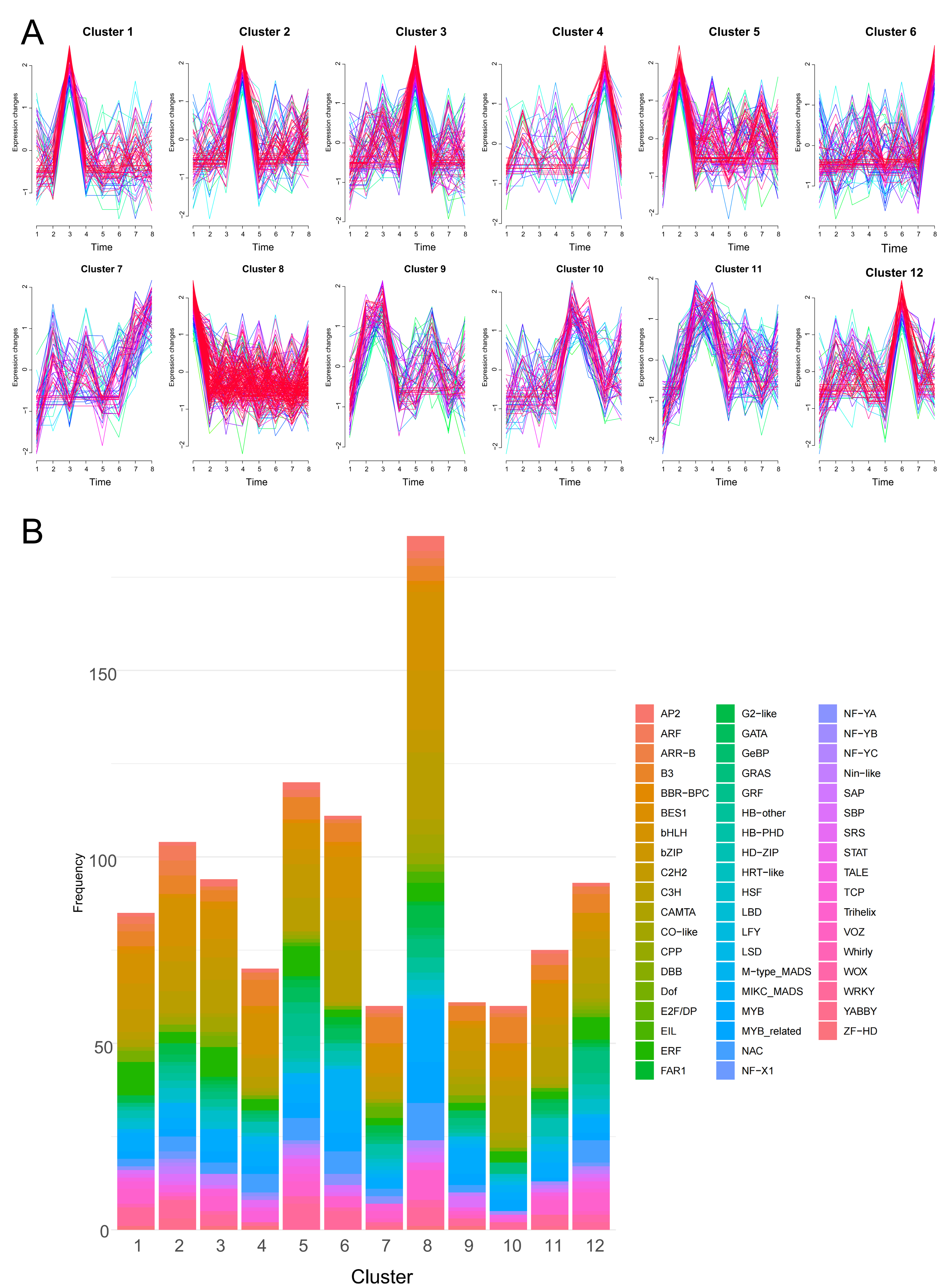
**

**Figure S10.** Gene expression patterns of transcription factors in spinach. A) A total of 1,119 identified TFs were grouped into twelve expression clusters, each showing distinct patterns during male and female inflorescence development. In the diagram, each line represents an individual gene: yellow and green lines indicate genes with low membership values, while red and purple lines indicate genes with high membership values. B) Bar plot showing the proportional distribution of different TF types identified by ST sequencing.

**
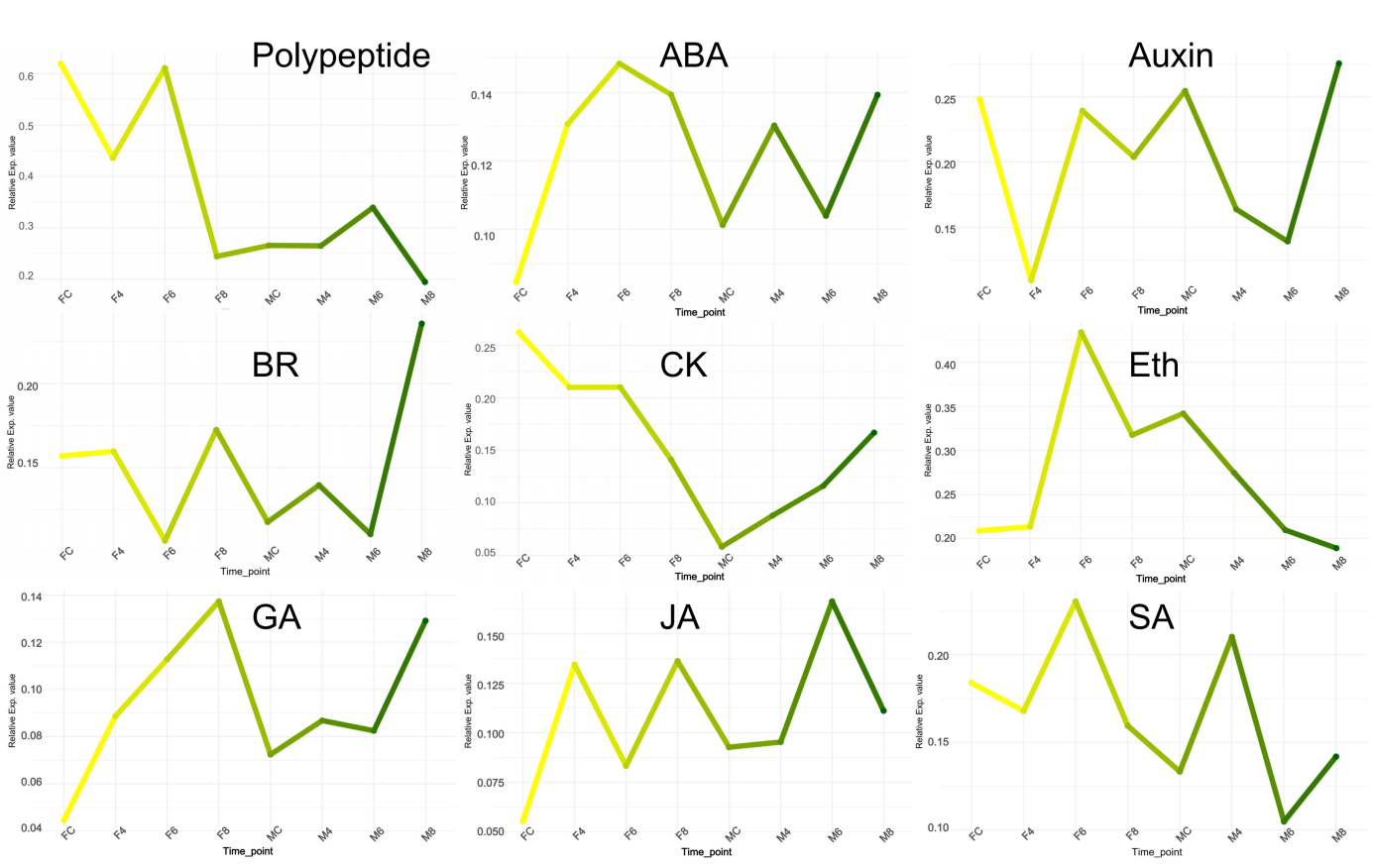
Figure S11.** Time-series expression patterns of hormone synthesis-related genes in spinach.The expression dynamics of genes involved in plant hormone synthesis were analyzed across developmental stages from cotyledon (FC/MC) to the eight-leaf stage (F8/M8). Relative expression levels were derived from ST data. These genes were subjected to time-series expression pattern analysis using the R package Mfuzz.


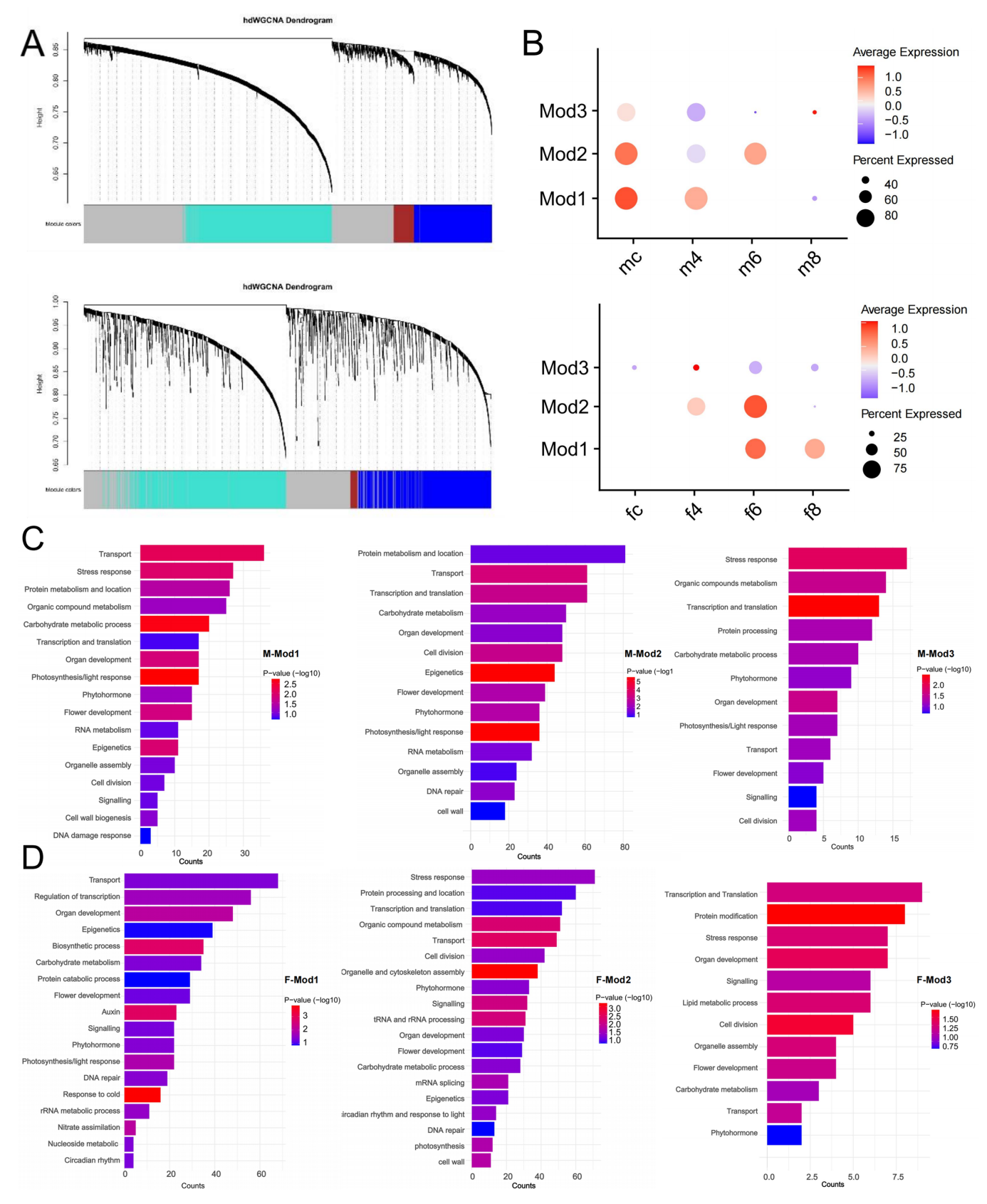


**Figure S12.** WGCNA analysis of the DEGs in developing female and male inflorescence.

1. Dendrogram of gene clustering. B) Expression pattern of each mode in developing female and male inflorescence. C-D) Enrichment of biological processes for the Co-expression modules in male and female samples, respectively.
